# Longitudinal analysis of social isolation effects on finger tapping in the Blursday database

**DOI:** 10.1038/s41598-023-38488-w

**Published:** 2023-07-12

**Authors:** Elisa M. Gallego Hiroyasu, Rodrigo Laje, Keishi Nomura, Ignacio Spiousas, Masamichi J. Hayashi, Yuko Yotsumoto

**Affiliations:** 1 Department of Psychology, Keio University, Tokyo, Japan; 2 Department of Science and Technology, University of Quilmes, Buenos Aires, Argentina; 3 Consejo Nacional de Investigaciones Científicas y Técnicas (CONICET), Buenos Aires, Argentina; 4 Department of Integrated Educational Sciences, The University of Tokyo, Tokyo, Japan; 5 Center for Information and Neural Networks (CiNet), Advanced ICT Research Institute, National Institute of Information and Communications Technology, Suita, Japan; 6 Graduate School of Frontier Biosciences, Osaka University, Suita, Japan; 7 Department of Life Sciences, The University of Tokyo, Tokyo, Japan

**Keywords:** Psychology, Human behaviour

## Abstract

The Blursday database is a collection of data obtained online from a longitudinal study where participants were asked to participate in several behavioral tasks and questionnaires during the COVID-19 pandemic from their homes. In this study, we analyzed the published data to explore (1) the longitudinal changes in temporal cognition observed from the data collected in the home-based setting (2), the effects of the voluntary quarantine measures implemented in Japan on temporal cognition, (3) whether the participant’s temporal cognition is altered by the change in their psychological state or their cognitive abilities, and (4) whether the effects of the quarantine measures depend on the age of the individual. Results show that confinement measures were good predictors for the performance in both spontaneous finger-tapping task and paced finger-tapping task, though these were dependent on the age of the participant. In addition, cognitive scores were good predictors of the performance in the paced finger-tapping task but not the spontaneous finger-tapping task. Overall, this study provides evidence suggesting changes in both psychological, cognitive, and temporal cognition during the pandemic on the Japanese population despite its voluntary measures to deal with the new situation.

## Introduction

In 2020, the COVID-19 pandemic hit worldwide in an unexpected and never-experienced manner. Across the globe, a large population of people got frightened of the new and rapid spread of coronavirus causing millions of infections and deaths worldwide. People were also threatened by the media showing several countries closing borders and going under strict lockdowns, meaning people were not allowed out of their homes.

The imposed quarantine measures have come with changes in our daily routines and schedules^1^. These changes in our daily routines also became the cause of countless stressors such as uncertainty about the future and the duration of quarantine, infection fears, inadequate supplies, inadequate information, financial loss and stigma^2,3^. As a result, numerous studies have also shown the detrimental effects the pandemic has had on our mental health^2,4^. Across the globe, lower levels of well-being as well as increased levels of stress, depression, anxiety, and loneliness were observed^2,5–13^ despite different governmental measures. Yet, the consequences of the quarantine measures were not limited to our psychological health as it has also been linked to a decline in cognitive abilities. Ingram and colleagues^14^ showed that across participants from their teens to their 70 s, attention, memory, and decision-making was altered, though these improved over 13 weeks as the social isolation measures were eased. Moreover, a decrease in working memory and prospective memory as a result of COVID-19 restrictions was shown among students^9^. Thus, the changes in our daily lifestyle, imposed by the fear of catching COVID-19 as well as the governmental measures severely impacted our mental and cognitive health.

The emotional state and cognitive abilities are greatly related to our temporal cognition and thus, people can sense time differently during the pandemic. Though some contradictory studies exist, it is shown that while anxiety can cause an underestimation of time, fear alone cannot^15^. Moreover, Mioni and colleagues^16^ showed that while anxious patients over-reproduce temporal intervals, depressed patients under-produce temporal intervals These differences were attributed to the reproduction of intervals being associated with memory and attention, in which the anxious patients suffer, while the depressed patients may suffer impairment in the motor action required to perform correctly in production tasks.

Indeed, psychological and cognitive factors that have been affected during the pandemic have been shown to distort our experience of time. In a study conducted during the first wave of the pandemic in the UK, Ogden^8^ showed that 80% of the participants had an altered sense of time. The differences in the perception of time were explained by the differences in the lived experience of the pandemic: positive emotional factors are shown to make time feel like it is going faster. In contrast, negative emotional aspects are shown to make time feel like it is going slower. Moreover, in France, Droit-Volet and colleagues^12^ showed that participants claimed that time was going by slower than usual, and the most reliable predictor of time was sadness and boredom. Other factors were related to the perception of time in different countries: age, well-being, stress, anxiety, depression, anger, fatigue, task load, and satisfaction with the quality of social interactions^8,12,13,17–20^. Not only was the perception of the passage of time affected, but also, it was shown that people were producing temporal intervals differently from previously^14^ and these changed as the effects of the pandemic lingered on. Thus, people all over the world experienced time in a different way during the COVID-19 pandemic.

Although it is clear that different governmental measures and the fear of the pandemic have altered people’s psychological health and cognitive abilities in different corners of the world, it is still unclear how much voluntary confinement can have on psychological and cognitive mental health. In Japan, the governmental measures were limited since the government could only give recommendations and abiding to strict lockdown measures (mandatory in the majority of other countries) was voluntary. Though schools and some workplaces were strongly recommended to close during the first wave of the pandemic, strict lockdowns were never imposed. People were still free to roam around the city to go out to eat and meet friends without any imposed fine like in some European countries. Yet, studies have shown that high levels of anxiety and depression were reported as well as psychological distress^21,22^. According to Yamamoto and colleagues^22^, psychological distress was significantly higher in May 2020, and these were influenced by increased levels of loneliness, anxiety, and sleeplessness, as well as decreased quality of social interactions, household economy, and difficulties in work or at school. Given that the psychological effects of the pandemic were clear in the Japanese population, it is possible that the perception of time is distorted as well. Hence, in this study, we investigate whether temporal cognition has been altered in the Japanese population and whether this distortion can be attributed to any psychological state and/or cognitive ability.

In this study, we utilized a subset of a worldwide study aimed at investigating the changes in our psychology and temporal cognition during the time of the pandemic lockdown around the world^23^ and specifically looked into two tasks performed by the Japanese population. In specific to Japan, participants residing in Tokyo participated in the study using an online platform, Gorilla Experiment Builder (www.gorilla.sc), shown to have good temporal accuracy^24,25^. Data were recorded in three “treatment” sessions: during the confinement in the first wave of COVID-19 (Session 1), one month after confinement (Session 2), and three months after confinement (Session 3). In addition, we ran a control session (approximately 15 months after Session 1). From this dataset, we specifically looked into the data from the spontaneous finger-tapping task and the paced finger-tapping task.

The reasons behind choosing these two tasks to investigate the effects of the pandemic on temporal cognition were related to how these two tasks compare to each other. Tapping at an isochronous rhythm, as was the case in these two tasks could be considered automatic for its rhythmic component. It has previously been shown that even infants can produce rhythmic, cyclic movements^26^. However, the task of tapping to a given pace could be cognitively demanding as the ability to adjust the motor timing to an external stimulus requires some effort and children are not born with this ability^27,28^. Guérin and colleagues^29^ found that while tapping at a faster tempo than that of the spontaneous motor tempo relied on bodily movement dynamics, tapping at a pace that is slower than one’s spontaneous motor tempo required cognitive control. Turgeon and Wing^30^ also found a dissociation between these two tasks by showing that while unpaced tapping is affected by age, paced tapping is not. Thus, by looking at the performance in the spontaneous finger-tapping task (at the pace of the spontaneous motor tempo—usually around 2 Hz) and the paced finger-tapping task (at the pace of one tap per second), we explore whether confinement and other psychological and cognitive variables had the same influence on these two similar but differing tasks.

In addition, we investigate whether there were differences in age in the effects of temporal cognition. From the start of the pandemic, older adult groups were said to form the vulnerable group, where COVID-19 posed a major risk of mortality^31^. In this sense, these were the subpopulation who had the greatest fear of catching COVID-19 and had to be separated from the rest. Thus, it was intuitive to think that their psychological and cognitive state would be impacted negatively in this population. Contrary to popular belief, however, the younger people had more concerns about the threat of COVID-19 and recorded higher levels of anxiety, sadness, frustration, and loneliness as well as lower positive affect^32^ than their older counterparts. Older people, on the other hand, have been shown to be more confident in the information they received, and have a positive attitude toward what was happening as well as restrictive measures imposed^33^. This was also true in Japan, where the baseline of loneliness, anxiety, and depression increased with age^34^ and in general, young, and middle-aged individuals suffered more from the pandemic in terms of mental health than the older adults^21^.

Hence, in this study, we (1) explore the effects of the voluntary quarantine measures implemented in Japan on temporal cognition, (2) explore whether the citizen’s temporal cognition is altered by the change in psychological state (anxiety, depression, or loneliness) or the cognitive abilities, and (3) explore how and whether the effects of the quarantine measures depend on the age of the individual.

## Methods

### Period of data collection

Data were collected longitudinally from participants residing in Tokyo on four different occasions at three different time points (Supplementary Fig. 1). The first session (S1) spanned the first state of emergency (April 7th, 2020–May 25th, 2020) between April 20th, 2020, and May 26th, 2020. Though lockdown was completely voluntary in Japan, public schools were mandated to close, companies were recommended to go online, and people in Tokyo were recommended to stay home as much as possible and reduce their shopping trips to three times a week. Even when the state of emergency was lifted, people were still asked to remain cautious. Session 2 (S2) and Session 3 (S3) were conducted approximately one month and three months after confinement, respectively (S2: June 8th, 2020, to July 7th, 2020; S3: September 23rd, 2020, to October 23rd, 2020). During this time, no strict recommendations were given by the governor besides asking people to avoid the “Three Cs” (1. Closed spaces with poor ventilation, 2. Crowded places with many people, and 3. Close-contact settings) and people slowly adapted to the “new normal”.

In addition, to explore whether there are observable differences in the measures during the pandemic and after the pandemic, we passed the same questionnaires and tasks to the new and naïve participants. This data set (Session Control, SC) was collected approximately 15 months after the first data collection (July 1st, 2021–July 21st, 2021). We hypothesized that by this time, participants were already habituated to the “new normal” lifestyle. Data is fully accessible online on the Blursday data server (https://dnacombo.shinyapps.io/TSDshiny/).

### Participants

Approximately 200 participants were recruited through an agency (Agekke Group), which filtered out those who had previously been diagnosed with psychiatric disorders or were taking medication. The ages were uniformly distributed between the ages of 20 and 70 (Supplementary Fig. 2). 108 participants concluded the entirety of S1 before the state of emergency was lifted and therefore added in this study. The ages of these participants ranged from 21 to 66 years old (Mean = 43.49, SD = 12.69), with 49% being female. Participants who did not complete the session were considered as wanting to drop out of the experiment and therefore did not proceed in the following sessions. By the end of S3, there were 95 participants (Mean = 44.39, SD = 12.69). For the control session, 100 participants uniformly distributed between the ages of 20–70 were recruited either through the same recruiting agency or through Twitter. 75 participants (Mean = 45.6, SD = 13.66, 46% female) completed the study and were, therefore, analyzed.

These participants were contacted through the recruiting agency or directly with the link to access the experiment on the Gorilla Experiment Builder (www.gorilla.sc)^25^. Once signed in, they were given written instructions and consent forms following the Declaration of Helsinki. This page informed the participants of the longitudinal nature of this study and their freedom to take a break between the tasks and questionnaires, as well as fully exit the experiment, whenever they felt the need. Those who completed each of the session were given monetary rewards for their participation. This protocol for Japan was approved by the institutional review board of The University of Tokyo (Japan).

### Instruments

The experiment was conducted using the Gorilla Experiment Builder (www.gorilla.sc) platform and participants were provided with a link to the website^25^. All questionnaires and tasks given to the participants are available on Gorilla Open Materials (https://app.gorilla.sc/openmaterials/286482). Each session took approximately two to three hours, but participants were free to take breaks and return another day as they saw fit. The session started with an explanation of the project and the informed consent, followed by the collection of demographic variables, and psychological questionnaires. Then, the participants proceeded to the multiple tasks included in the Time Social Distancing study^23^. These tasks included cognitive tasks (such as the three-back task) and temporal tasks. Each of the tasks of interest in our study appeared three times (which we term as Run) throughout the session, interweaved among the other different temporal tasks not included in our study. Given that each session was long, it was not necessary for participants to finish all the runs on the same day.

#### Confinement measure

Due to our interest in the possible effect of confinement on the Japanese population, we also included the movement of people in transit stations as recorded by the Google Mobility report^35^. Since for each participant, we have one timing task variable per session (mean of all runs), each subject was attributed one confinement measure which was the seven-day centered moving average of the transit score of the Google Mobility report from the day the run two of the temporal task was performed. Taking the seven-day centered moving average of the transit score would account for differences in time in which the participant took part in the three runs of the temporal tasks, as well as account for the delay for the degree of confinement in society to have an effect on the subject, and the acute differences in the scores during the weekend (Supplementary Fig. 1).

#### Psychological measures

To measure the psychological state of the participants and to see whether their levels of anxiety, depression, and loneliness were affected by the COVID lockdown, participants were asked to answer a few questionnaires. The following questionnaires were answered once toward the beginning of each session before any of the temporal tasks were conducted.

##### Hospital anxiety and depression scale (HADS)

The anxiety and depression scores of the participants were indexed using the Japanese version^36,37^ of the original Hospital Anxiety and Depression Scale^38,39^. Though this scale was primarily developed for clinical use, recently, it has been validated for the general population^40,41^ and used for many studies investigating how COVID-19 may have affected anxiety and depression levels of the people^42,43^. This questionnaire includes seven items for anxiety and another seven items for depression. Each of their scores ranges from a total of 0 to 21 where the higher score depicts severe depression and anxiety.

##### UCLA loneliness scale

Loneliness measures of the participants during and after confinement were indexed using the UCLA loneliness scale^44,45^*.* This questionnaire consists of 20 questions to assess how satisfied one is with their social interactions. A total score can range from 0 to 60 where the highest score depicts severe loneliness. The reliability of this scale to the general population has been reported^46^. To match the English version used for this international project, the questionnaires used here were adapted from the Japanese version of this questionnaire^47^.

#### Cognitive measures

To measure the cognitive capabilities of the participants and to see whether cognitive functions, especially that of working memory, were affected by the COVID lockdown, participants were asked to perform a three-back task. This task requires participants to observe a sequence of letters on the screen and answer whether the displayed letter was identical to the letter that had appeared three letters ago. Each run consisted of four blocks with either 25 or 50 letters in each block. Participants completed three runs per session.

#### Temporal cognition measures

##### Spontaneous finger-tapping task

This task requires participants to tap by pressing the space button at their comfortable speed when the start cue appears. Unbeknownst to the participants, participants tapped for a duration of 1 min.

Considering the variety of responses found in this task, we cleaned the data using three consecutive criteria. First, we calculated the median inter-tap interval (ITI) for every trial of every participant and filtered out single ITIs that were below 1 ms and over 5 s. These were very likely due to system glitches or the participant not responding (that can be related to the *error outliers* in the nomenclature of Leys and colleagues^48^. Second, we discarded the full trial if it had more than 30 ITIs under 50 ms (most likely due to the participant keeping the key pressed down; *error outliers*^48^). Finally, we detected and removed outliers from the resulting trial median ITI distribution (*random outliers*^48^) using the robust median absolute deviation with a threshold of three using the *outliers_mad* function in the *Routliers* package for R^49^. After all the exclusion criteria were applied 111 trials were discarded from a total of 1123 (9.9%).

##### Paced finger-tapping task

The visual synchronization-continuation task involves two sections that follow each other: the synchronization and continuation phases. In the synchronization phase, participants had to tap the spacebar with the appearance of the visual stimuli. The interstimulus interval was one second. In the continuation phase, participants had to maintain the rhythm from the synchronization phase and continue tapping at the same rate. While embedded in the same task, both synchronization and continuation are supported by different neural mechanisms^50^ and one can be altered in one but not the other^51^. More specifically, the act of synchronizing to the beat is thought to evoke error-correcting mechanisms^52,53^, only the internal time-keeper controls and aids the motor aspect of the tapping during continuation. One trial lasted two minutes in total with each phase lasting one minute. The duration was not mentioned to the participants.

Given the complexity of this task when performed online with no supervision, there was a multitude of responses indicative of a misunderstanding of instructions (*error outliers*^48^). For instance, some participants were clearly tapping OutSync during InSync trials (their mean asynchrony was consistently around 0.5 times the inter-stimulus interval (ISI), which is much longer than typical asynchronies in the literature), and vice versa. Other participants were clearly drifting away (i.e. keeping their own tempo regardless of the stimuli). Still, others reacted to every stimulus instead of synchronizing to the sequence (their asynchronies were consistently 250 ms after the stimuli). Unlike an in-person laboratory setting where the experimenter can help the participant understand the task and correct misunderstandings with a single instruction, here we have no other way but to discard all invalid trials afterward.

In addition, there were also the more usual random outliers, such as very large or very small random values in a distribution^48^. To remove these participants, specific exclusion criteria were applied to both the synchronization and the continuation phase. If an outlier was detected in the synchronization phase, then we removed the whole trial (synchronization and continuation). If an outlier was detected only in the continuation phase of a trial, only the continuation phase was removed. After all the exclusion criteria were applied 393 trials were discarded from a total of 1126 (35%). Session 1 went from 324 to 183 trials (141 trials excluded), session 2 went from 297 to 189 trials (108 trials excluded), session 3 went from 282 to 205 trials (77 trials excluded) and the control session went from 223 to 156 trials (67 trials excluded). More details of the exclusion criteria can be observed in the supplementary materials (Supplementary Material 1).

From this task, two measures were obtained. First, from the synchronization phase, the measure of asynchrony was obtained as the difference between the occurrence times of every response (Rn) and its corresponding stimulus (Sn) in a trial: ${Asy}_{n} = {R}_{n}-{S}_{n}$. Asynchronies can be either positive or negative and have a mean value that is usually negative in a laboratory setting (minus a few tens of milliseconds), meaning that responses are commonly in advance of the stimuli (negative mean asynchrony, NMA)^54^. The mean asynchrony in a platform like Gorilla, however, could be closer to zero and even positive due to larger system latencies. Asynchronies are commonly much shorter than reaction time^55^ and participants can maintain a constant average asynchrony thanks to an error correction mechanism that takes in the past performance and predicts the occurrence time of the next stimulus^53,56^. The second measure is the median ITI in the continuation phase of each trial. This measure, also termed reproduced interval, is the length of the interval that the participants were reproducing when asked to reproduce one second.

### Statistical analysis

First, we investigated the relationships between the change in the temporal interval produced when tapping at a comfortable speed and the other factors including the demographic variable of Age, the measure of confinement, the psychological variables of Anxiety, Depression, and Loneliness as well as the cognitive score (performance in the 3-back). The degree of change during the COVID-19 pandemic was analyzed by transforming the score obtained for the psychological and cognitive measures, as well as the confinement measure, as follows: For all these variables, we subtracted the performance or score in S3 (“the new normal”) from the performance or score of S1, S2, and S3. In other words, the scores in S1 and S2 would reflect the measures in reference to the “new normal” and the performance in S3 is illustrated in the graphs of the results with the value of zero. For example, in the measure of confinement, which is the change in the mobility scores of the people in transit stations, zero is the value where most mobility was observed, as it was the case in S3 and the smaller the value, the less mobility was observed and therefore, the more confined. The change in temporal production (ITI in the spontaneous finger-tapping task) as well as the asynchrony and the reproduced interval of the paced finger-tapping task (Asynchrony and ITI in the synchronization-continuation phase, respectively) were also calculated in the same manner for the purpose of the correlation analysis. Analysis was conducted using JASP^57^.

Next, to examine which factors can predict the performances in the spontaneous finger-tapping task and the paced finger-tapping task, we conducted Bayesian Multilevel modeling with the mean inter-tap interval as the dependent variable in the free spontaneous finger-tapping task and the asynchrony of the synchronization phase and the inter-tap interval of the continuation phase as the dependent variables in the paced finger-tapping task. For each of the three temporal measures, 42 models, each varying in the factors considered, were compared. Models included in the comparison can be seen on supplementary materials (Supplementary Table 1). For the purpose of modeling, the variable of Age was centered on the mean and the other independent variable (confinement, psychological, cognitive measure) were transformed in reference to the “new normal”. For all models, we assumed that the intercept of the temporal measures was dependent on each participant (random effect of participant). When the models included psychological or cognitive variables, we assumed that intercepts and slopes for these scores for each participant were random effects.

Model comparisons were conducted using the global level of the Watanabe Akaike information criterion (WAICg) for the gaussian model family as implemented in the {glmmstan} R package. In contrast to the regular WAIC, this comparison method allowed us to predict data from new participants for the time points we have, rather than predict data from existing participants at new timepoints as it is in the case of regular WAIC. The interpretation of the coefficients of the winner model is of a more exploratory character and only the coefficients included in the top two models are discussed. Figures obtained from the model comparison results are illustrated using the {brms} R package.

Furthermore, to differentiate confinement effects and possible practice effects in the longitudinal data, we conducted a Bayesian T-test using JASP^57^ on all the factors mentioned above. For each temporal measure, we compared the score and performance between S1 and SC, and later (though not pre-registered), we did the same for S3 and SC since we realized that this could clearly show the effects of practice effects.

The study was pre-registered on July 22nd of 2022, using the Open Science Framework (https://osf.io/f953y).

## Results

### Relationship between the psychological, cognitive and temporal factors over time

To investigate the relationships between the changes in the psychological, cognitive, and temporal factors during the COVID-19 pandemic, correlation analysis was conducted using the transformed variables of Age, Anxiety, Depression, Loneliness, Cognitive score, and Confinement of all the participants that took part in this study (Supplementary Table 2). The findings of the present study suggested that a reduction in anxiety over time was associated with a decrease in depression over time, which was supported by strong evidence for the positive correlation between Anxiety and Depression (r = 0.311, BF > 1000). Additionally, there was a trend suggesting that a decrease in anxiety over time was correlated with the decrease in loneliness over time, as evidenced by a positive correlation between Anxiety and Loneliness (r = 0.143, BF = 1.351). Moreover, the results indicated that a decrease in depression over time was associated with an improvement in cognitive functioning, as evidenced by a negative correlation between Depression and Cognitive score (r =  − 0.163, BF = 3.016). However, no other psychological and cognitive variables were correlated with each other (BF < 1).

Results also indicated that changes in confinement measures were significantly associated with changes in both cognitive and psychological outcomes. Specifically, a strong positive correlation was found between Confinement and Cognitive score (r = 0.425, BF > 1000), suggesting that as the confinement measures were being relaxed, participants performed better in the cognitive task. Furthermore, as confinement measures became less strict and participants moved around more, they reported feeling less depressed over time as shown by the substantial evidence supported a negative correlation between Confinement and Depression (r =  − 0.229, BF > 100). There was also substantial evidence supporting the idea that people felt less lonely during the strict confinement periods as shown by the correlation between Confinement and Loneliness (r = 0.149, BF = 1.728). Taken together, these findings suggested that changes in confinement measures were associated with changes in depression, loneliness, and cognitive scores in the studied participant sample.

Moreover, we investigated whether the temporal variables obtained in the change in spontaneous finger-tapping and paced finger-tapping tasks were correlated with any of the transformed variables of Age, Anxiety, Depression, Loneliness and Cognitive score using only the data of those participants included in the temporal performance analysis. In terms of the relationships between temporal performance of the spontaneous finger-tapping task and the other factors, there was evidence for the correlation between the changes in the interval produced with the Cognitive score (r = 0.184, BF = 7.988) and with Confinement (r = 0.240, BF > 100). The positive correlation with the change in produced intervals illustrated the idea that as people got habituated to the new normal and got better at the cognitive task, their reproduced intervals got longer, in other words, tapped at a slower pace. However, no evidence was obtained for the correlation between the psychological, cognitive or confinement measure on the two measures of the paced finger-tapping task.

### Changes across sessions

#### Participant demographics and confinement values across sessions

Though this section of the analysis was not preregistered, throughout the duration of the longitudinal study, participants were allowed to freely drop out of the study and therefore, we confirmed using the analysis here that there was no difference in age in the participants that remained throughout the three sessions: S1 compared to S2 (BF < 1; S1: N = 108, Mean = 43.389, SD = 12.571, 95% Credible Interval (CrI) [40.991, 45.787]; S2: N = 99, Mean = 44.182, SD = 12.483, 95% CrI [41.692, 46.671]), S2 compared to S3 (BF < 1; S3: N = 94, Mean = 44.614, SD = 13.392, 95% CrI [41.421, 47.807]), and S1 compared to S3 (BF < 1). Hence, it could safely be said that the changes in different variables studied between these sessions is not likely to be caused by differences in the ages of the participants.

Moreover, it was also confirmed that the level of confinement changed as data was being collected in the longitudinal study. When Bayesian Samples T-test was run, extremely strong evidence was observed suggesting change in confinement scores across the three sessions: S1 compared to S2 (BF > 1000, S1: N = 108, Mean =  − 58.042, SD = 6.425, 95% CrI [− 59.268, − 56.817], S2: N = 99, Mean =  − 33.740, SD = 3.116, 95% CrI [− 34.362, − 33.119]), S2 compared to S3 (BF > 1000; S3: N = 94, Mean =  − 27.733, SD = 2.815, CrI [− 28.309, − 27.156]), and S1 compared to S3 (BF < 1). Therefore, it could be said that as participants were taking part in the experiment, mobility in transit stations was slowly approaching normality as compared to the baseline. Moreover, this comes to show that while confinement was purely voluntary, the Japanese population did in fact stay at home during the midst of the COVID-19 pandemic fears in May of 2020.

#### A look into the longitudinal data (S1 vs. S3)

To get a clear overview of how the participants performed in the longitudinal data, a simple Bayesian T-test was conducted, comparing the multiple factors of the raw scores of anxiety, depression, loneliness, and cognitive score between S1 and S3 of all participants (Supplementary Table 3). The Bayesian Samples T-test results show that participants performed better in S3 (N = 93, Mean = 81.043, SD = 7.959, 95% CrI [79.404, 82.683]) compared to S1 (N = 107, Mean = 76.367, SD = 7.901, 95% CrI [74.853, 77.882]) in the cognitive task (BF > 100). Moreover, there was anecdotal evidence (BF = 1.416) suggesting greater depression scores in S1 (N = 108, Mean = 9.019, SD = 3.724, 95% CrI [8.308, 9.729]) compared to S3 (N = 95, Mean = 7.863, SD = 3.791, 95% CrI [7.091, 8.636]).

In addition, we also compared the temporal data between the two sessions using the data of only the participants included in the temporal analysis. Produced intervals were shorter in S1 (N = 108, Mean = 676.347, SD = 309.705, 95% CrI [616.701, 735.992]) compared to S3 (N = 94, Mean = 764.215, SD = 279.212, 95% CrI [707.027, 821.403]) as shown by anecdotal evidence (BF = 1.190). All other factors had evidence for no difference (BF < 1).

#### Significant predictors for temporal performance

To explore (1) whether the trend in differing temporal performance can be explained by how age affected the lived experience of confinement, (2) whether there is a difference in age in relation to how different psychological effects and cognitive scores may impact temporal performance, and (3) whether and how the overall relationship between the confinement level and the temporal productions depends on the psychological variables or cognitive variables, we conducted Bayesian Hierarchical Modeling with each of the temporal measures. For each of the following, 42 models were compared using WAICg and as preregistered, the top two models are discussed. The WAIC and WAICg for all other models can be found in the supplementary materials (Spontaneous finger-tapping task, Supplementary Table 4; Paced finger-tapping task (Asyn), Supplementary Table 5; Paced finger-tapping task (ITI), Supplementary Table 6) and on OSF. Note here, that for this section and for the purpose of modeling, the significant predictors of Confinement and Cognitive score refers to the transformed variables, where all raw scores were subtracted from the recorded score in S3.

##### Spontaneous finger-tapping task

Results for the model comparisons in the produced interval of the spontaneous finger-tapping task revealed the top models to be: (1) the model including the interaction effect of Age and Confinement, and (2) the model including the main effect of Age as well as the interaction effect of Age and Confinement.

**Figure 1:**
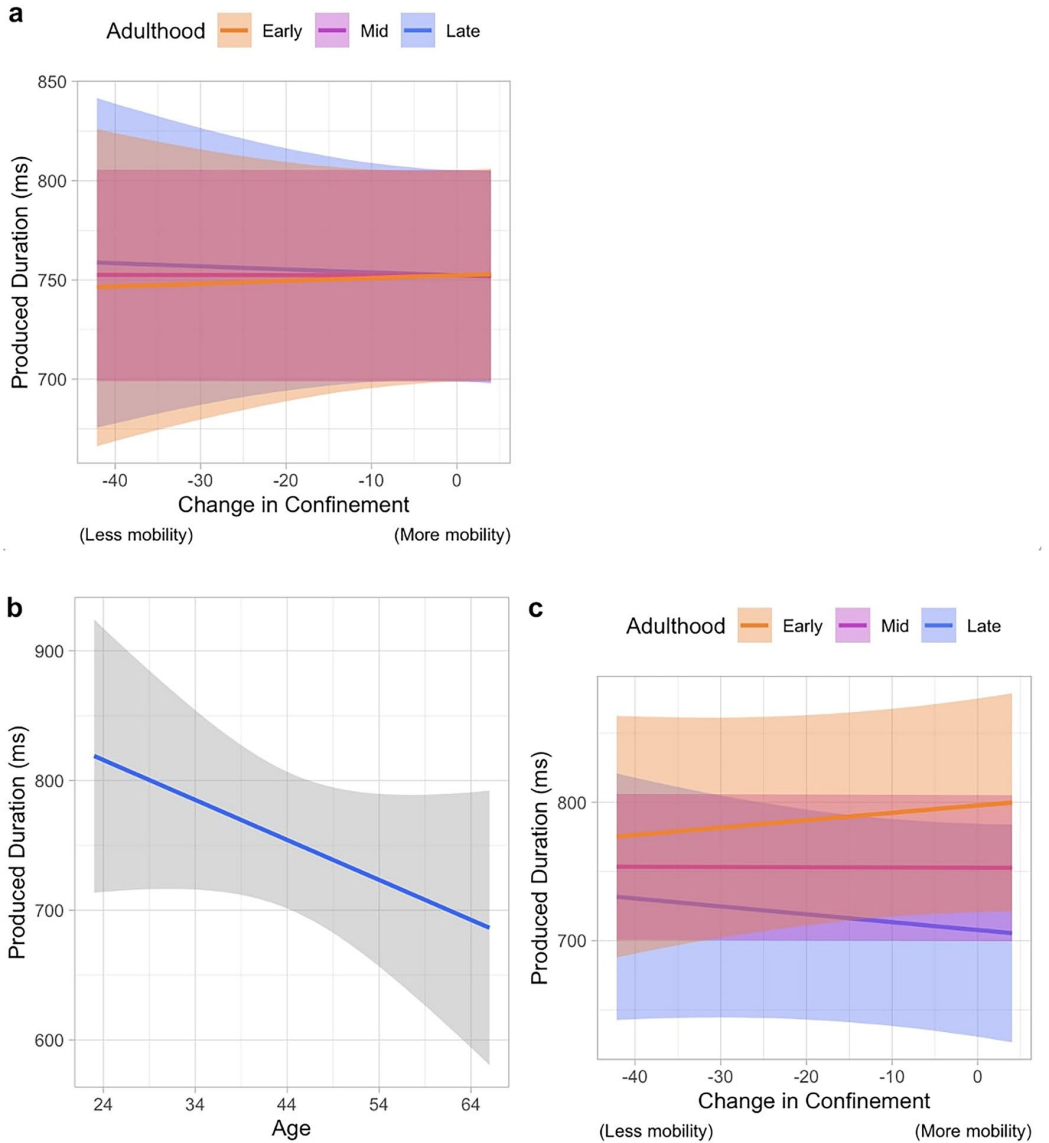
The parameters in (**a**) the best model, and (**b**, **c**) the second-best model for the produced interval in the spontaneous finger-tapping task. The best model includes the coefficient of the interaction between (**a**) the interaction between Confinement and Age. For the second-best model, the coefficients included are the (**b**) main effect of Age, and (**c**) the interaction between Confinement and Age. The orange, magenta and blue color depicts the participants in the early, mid, and late stage of adulthood, respectively. Note that the x-axis values for the transformed variables are relative to the measures of S3. Therefore, in the context of “Change in Confinement”, the value of zero represents the baseline mobility observed in S3, which corresponds to a period when mobility had returned to normal post first wave of pandemic (less confined). Additionally, smaller values on the x-axis indicate reduced mobility (increased confinement).

The best model includes the coefficient of confinement and age (WAICg = 4034.802, R2 = 63.412%, Supplementary Table 4) where the mean produced interval was approximately 700 ms (ß = 698.785, SD = 27.745, 95% CrI = [642.510, 751.581]). As shown in Fig. 1a, there was an effect of the interaction Confinement*Age (ß = -0.014, SD = 0.000, 95% CrI = [− 0.131, − 0.104]) indicating that for participants in the later stage of adulthood, the more the mobility resumed to the new normal, their produced interval got shorter while for those in the early stage of adulthood, the more the lifestyle resumed to normal, the produced interval got longer. However, looking at the posterior samples extracted, we can see that only 57.9% of the distribution for the coefficient of the interaction of Confinement*Age is below zero meaning that this effect is associated with large uncertainty.

The model that comes to a close second is one that includes the main effect of Age, and the interaction of Age*Confinement (WAICg = 4036.394, R2 = 0.635). This model shows that the subjects tapped at a mean interval of a bit over 700 ms (ß = 701.047, SD = 27.710, CrI [644.888, 753.625]). The main effect of Age (Fig. 1b) suggests that there is a 94.271% probability that as participants got older, the more they tended to have shorter intervals, meaning tapped at a faster pace (ß =  − 3.442, SD = 0.008, CrI[− 7.993, 1.134]). Having included the parameter of Age, the effect of Confinement*Age (Fig. 1c) differs, such that it indicates a trend whereas the life resumes to normal, the population in the early stage of adulthood tends to approach the true value and therefore produce longer intervals than during confinement, while those in the later stage of adulthood deviated further from the true value, causing a greater under-reproduction of the one-second interval (ß =  − 0.044, SD = 0.063, CrI [− 0.166, 0.080]). Though there is still a considerable amount of uncertainty in the interaction effect, the trend suggested in this second-best model was supported by 75.919%.

##### Paced finger-tapping task

For the paced finger-tapping task, we considered two dependent variables: the asynchrony in the tap during the synchronization phase of the task, and the inter-tap interval (sometimes called the reproduced interval) in the continuation phase of the task.

To start with, results for the model comparisons in the asynchrony of the tap during stimulus synchronization revealed the top models to be: (1) the model including the main effect of Confinement, Age, and Cognitive score, and (2) the model including the main effect of Confinement, Age, Cognitive score as well as the interaction of Age*Confinement.

**Figure 2:**
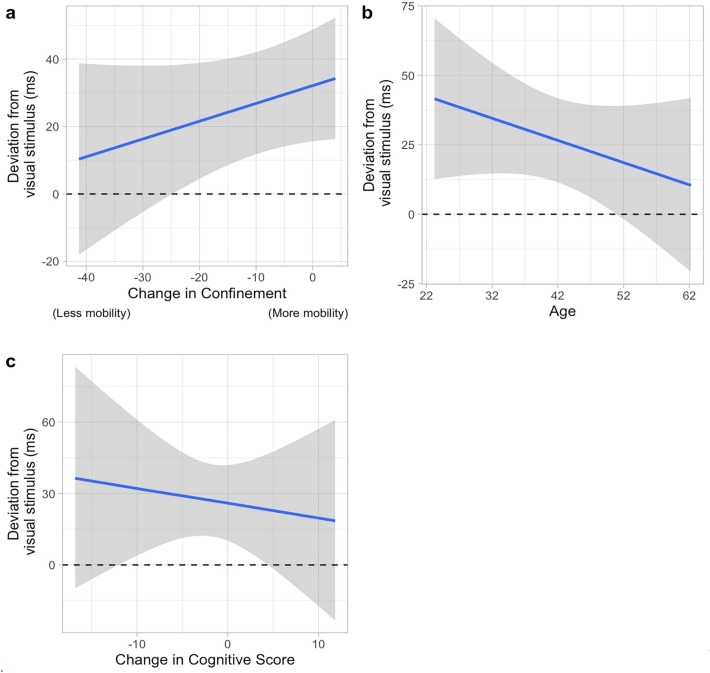
The parameters in the best model for the asynchronicity of the tap in the synchronization phase of the paced finger-tapping task. The horizontal line at zero indicates perfect synchronicity. This model includes (**a**) the main effect of Confinement, (**b**) the main effect of Age, and (**c**) the main effect of the Cognitive score.

The best model which included Confinement, Age, and Cognitive score as significant predictors (WAICg = 1741.754, R2 = 0.609, Supplementary Table 5) showed that people tended to tap at a mean of 30 ms after the appearance of visual stimulus (ß = 30.768, SD = 8.101, 95% CrI [14.858, 46.671]). This is indicative of participants overestimating the occurrence time of the coming stimulus in the synchronization phase, though an asynchrony of 30 ms is consistent with known system’s latencies^25^. In terms of the effect of confinement (Fig. 2a), 91.81% of the predicted samples revealed that participants deviated more from the occurrence time of the stimulus as confinement measures were being lifted (ß = 0.537, SD = 0.376, 95% CrI [− 0.199, 1.273]). The negative coefficient of Age (ß =  − 0.791, SD = 0.672, 95% CrI [− 2.105, 0.531]) indicated, and in accordance with 88.203% of the predicted samples, that as the participants got older, they tended to be better synchronized to the visual stimuli (Fig. 2b). Cognitive score (ß = − 0.791, SD = 0.672, 95% CrI [− 2.105, 0.531]) also reveals that the magnitude of asynchrony decreased with higher cognitive performance (Fig. 2c). However, this was only true for 66.51% of the predicted samples and thus, we see that there is great uncertainty here.

**Figure 3:**
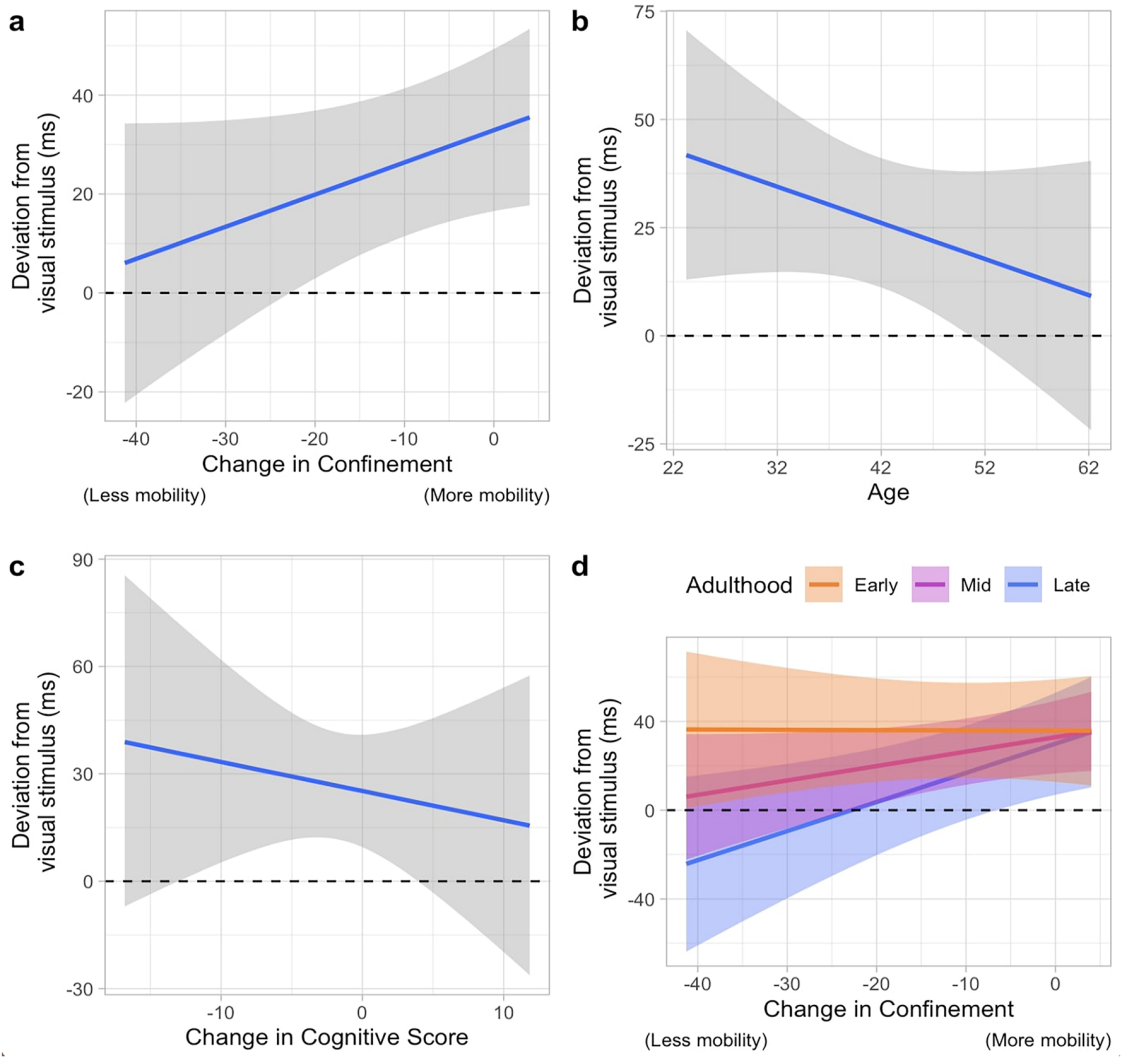
The parameters in the second-best model for the asynchronicity of the tap in the synchronization phase of the paced finger-tapping task. The horizontal line at zero indicates perfect synchronicity. This model includes (**a**) the main effect of Confinement, (**b**) the main effect of Age, (**c**) the main effect of the Cognitive score, and (**d**) the interaction effect of Confinement and Age. The orange, magenta and blue color depicts the participants in the early, mid, and late stage of adulthood, respectively.

The second-best model (WAICg = 1742.780, R2 = 0.623) also has a mean asynchrony of approximately 30 ms (ß = 31.505, SD = 8.029, 95% CrI = [15.656, 47.309]) with the three coefficients showing the same trend as in the best model (Fig. 3a–c): Age (ß =  − 0.278, SD = 0.706, 95% CrI[− 1.661,1.113]), Confinement (ß = 0.681, SD = 0.377, 95% CrI [− 0.058, 1.421]) and Cognitive score (ß =  − 0.858, SD = 1.427, 95% CrI [− 3.671, 1.935]). The posterior samples show that 88.203%, 91.807%, and 66.506% of Age, Confinement, and Cognitive scores are consistent with these trends. In addition to these parameters, the inclusion of the interaction effect of Confinement*Age shows that while the participants in the early stage of adulthood did not differ much in the measure of asynchrony throughout the three months of data collection, those in the later stage of adulthood did, going from predicting the arrival of the visual stimulus in an early fashion to a late fashion (Fig. 3d). This trend was observed for 98.461% of the posterior samples of this model.

As for the results in the model comparison of the reproduced interval (inter-tap interval) of the continuation phase of the paced finger-tapping task, the top two models are the same as in the reproduced intervals: (1) the model including the main effect of Age, Confinement, Cognitive score, and (2) the model including the interaction of Age*Confinement apart from those parameters in the best model.

**Figure 4:**
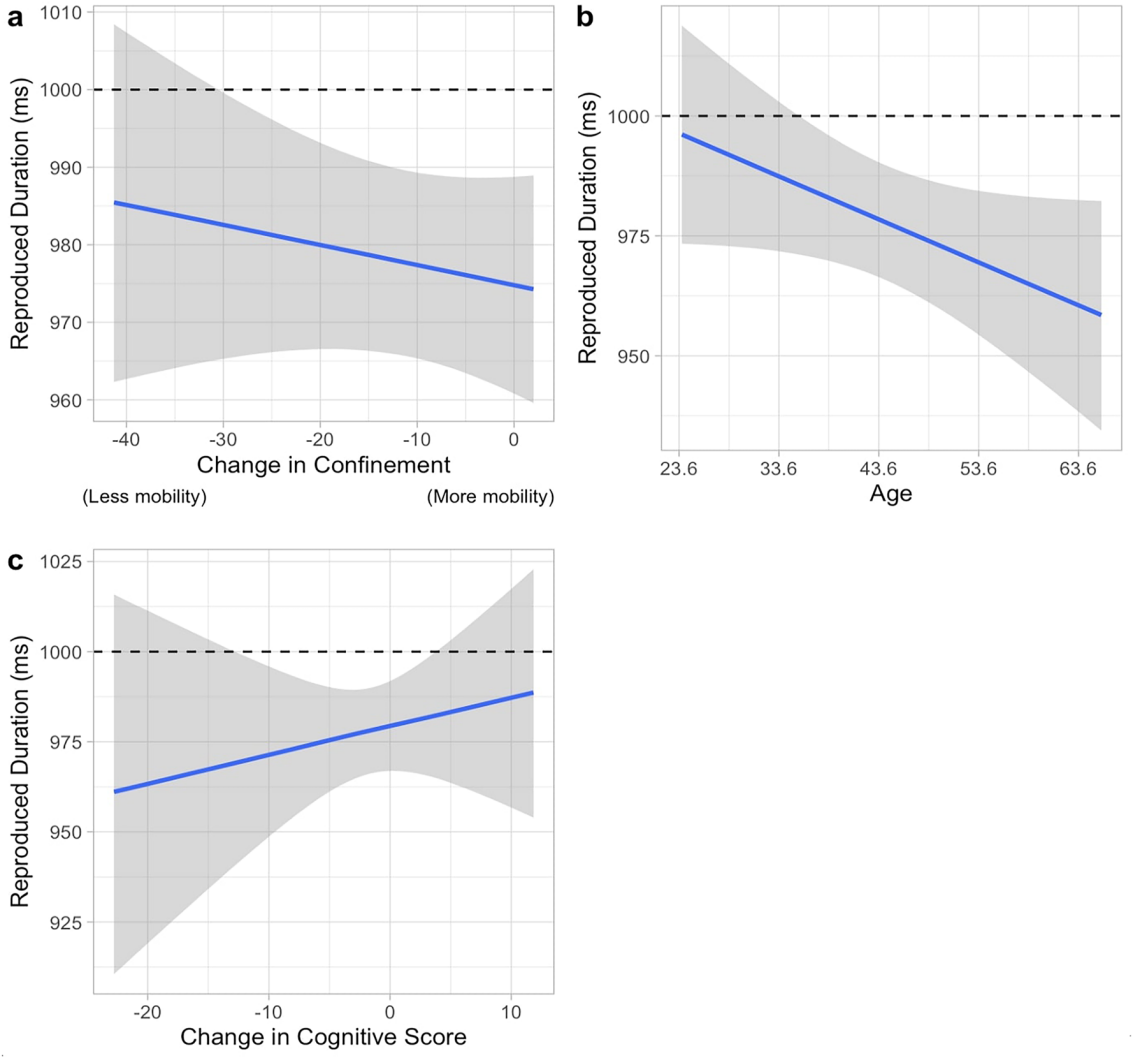
The parameters in the best model for the reproduced interval (ITI) of the paced finger-tapping task. The horizontal line at 1000 ms indicates the temporal interval that the participants were asked to reproduce. This model includes (**a**) the main effect of Confinement. (**b**) the main effect of Age, and (**c**) the main effect of the Cognitive score.

The best model (WAICg = 2631.352, R2 = 0.451, Supplementary Table 6) includes the coefficient of confinement, age where the mean produced interval was 973 ms (ß = 972.971, SD = 6.603, 95%CrI = [959.837, 985.800]). As shown in Fig. 4a, results showed that the trend was indicative of shorter intervals being produced as the confinement measures were being lifted (ß =  − 0.351, SD = 0.330, 95% CrI = [− 1.000, 0.291]), in a way that participants were tapping at a faster pace than the one initially marked. The effect of Age (ß =  − 0.893, SD = 0.479, 95% CrI = [− 1.833, 0.049]) indicates that there is a 96.82% probability that as age increased, shorter intervals were being reproduced as shown in Fig. 4b. The coefficient of Cognitive performance (ß =  − 0.887, SD = 1.174, 95% CrI = [− 1.477, 3.130]) was also significant in the performance of the top model, illustrating that as the performance in this task increased, the closer the produced interval approached 1000 ms (Fig. 4c). It is to note, however, that the posterior samples of confinement and the cognitive score show a great deal of uncertainty given that 74.174% and 78.301% of the posterior samples of Cognitive score and Confinement, respectively, were in accordance with the trend.

**Figure 5:**
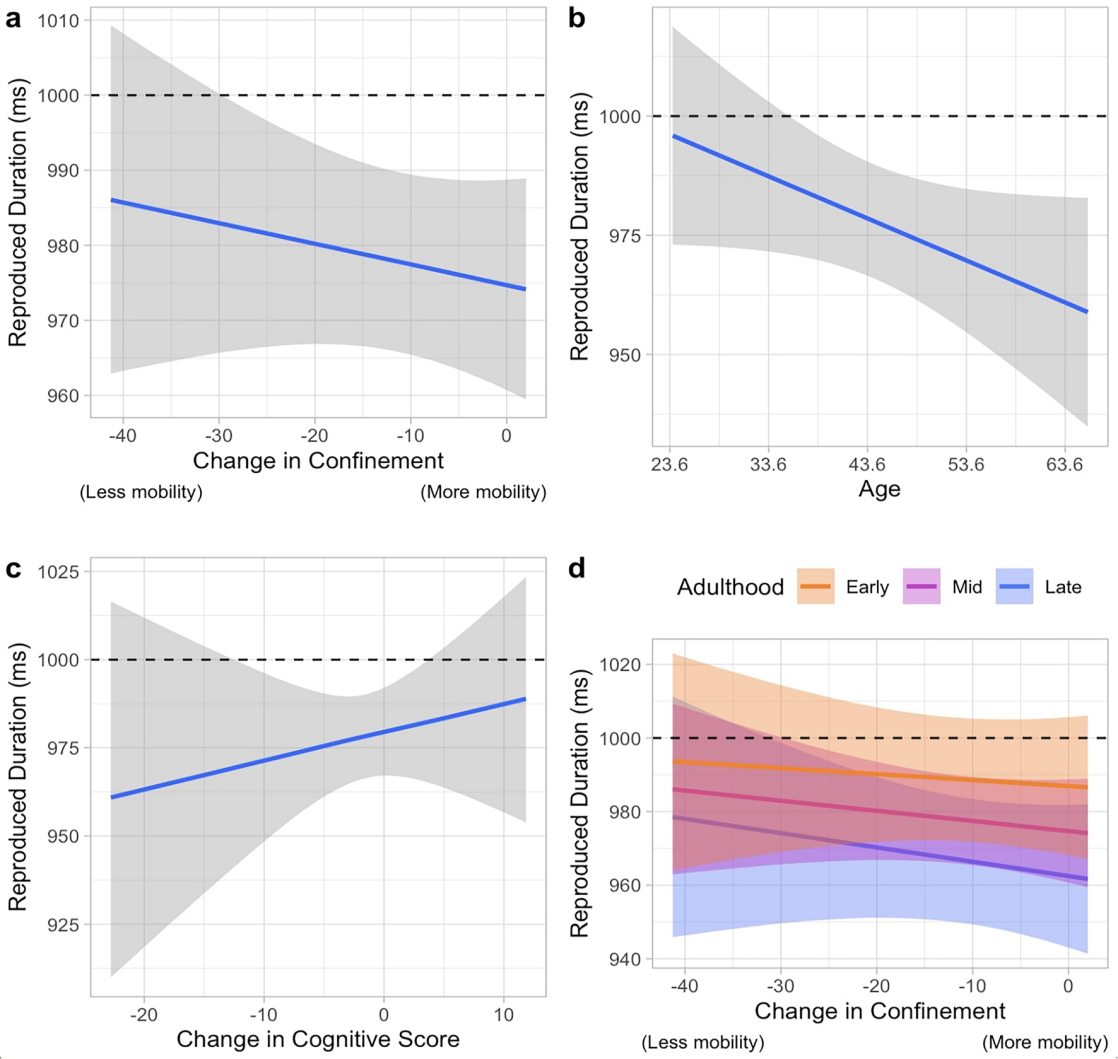
The parameters in the second-best model for the reproduced interval of the paced finger-tapping task. The horizontal line at 1000 ms indicates the temporal interval that the participants were asked to reproduce. This model includes (**a**) the main effect of Confinement, (**b**) the main effect of Age, (**c**) the main effect of the Cognitive score, and (**d**) the interaction effect of the Confinement and Age. The orange, magenta and blue color depicts the participants in the early, mid, and late stage of adulthood, respectively.

The second-best model is one that includes the interaction of Age*Confinement in addition to the predictors in the first model (WAICg = 2632.642, R2 = 0.453). This model shows that the subjects tapped at a mean interval of 973 ms (ß = 972.901, SD = 6.606, 95% CrI [959.762, 985.752]). Trends of the coefficients of Age (ß =  − 0.975, SD = 0.527, 95% CrI [− 2.009, 0.060]), Confinement (ß =  − 0.364, SD = 0.331, 95% CrI [− 1.014, 0.283]) and Cognitive score (ß = 0.912, SD = 1.176, 95% CrI [− 1.466, 3.163]), were identical to that of the best model (Fig. 5a–c) with 96.798%, 79.052% and 74.272% of the predicted data showing this trend, respectively. As for the interaction between Confinement*Age, 66.105% of the predicted data showed a trend where all ages reproduced increasingly shorter intervals than those first paced during the confinement period but with the change the participants in the later stage of adulthood being larger than those in the earlier stage of adulthood (Fig. 5d).

### A look into the confinement effect (S1 vs. SC)

Given that the longitudinal data may confound confinement and practice effects, data was also collected from naïve participants (SC) and compared to the psychological, cognitive state, and temporal performance of those from S1. While these two sessions involve different participant samples, there was no evidence suggesting a difference in Age among these two groups (BF = 0.198, S1: N = 108, Mean = 43.382, SD = 12.571, 95% CrI [40.991, 45.787]; SC: N = 70, Mean = 44.614, SD = 13.392, 95% CrI [41.421, 47.807]). Confinement measures of these two sessions, however, differed with extreme evidence (BF > 1000), such that people moved around more during the new normal (Mean =  − 28.397, SD = 0.602, 95% CrI [− 28.538, − 28.257]) than when the lockdown was first imposed (Mean =  − 58.042, SD = 6.425, 95% CrI [− 59.268, − 56.817]).

According to the results from the Bayesian Samples T-test (Supplementary Table 7), there is strong evidence to suggest that there is a difference in the depression level in the sample collected during the start of the pandemic to the participants who took part in the data collection during the new normal (BF > 10). Specifically, participants in S1 had higher depression scores (N = 108, Mean = 9.019, SD = 3.724, 95% CrI [8.308, 9.729]) compared to the naïve participants in the new normal (N = 72, Mean = 7.361, SD = 3.186, 95% CrI [6.612, 8.110]). Moreover, there was substantial evidence for the difference in cognitive scores (BF = 6.749) suggesting that the participants living in the new normal obtained higher scores (N = 73, Mean = 79.828, SD = 8.145, 95% CrI [77.928, 81.729]) than the participants in May of 2020 (N = 107, Mean = 76.367, SD = 7.901, 95% CrI [74.853, 77.882]). All other factors, including those of the temporal variables had evidence for no difference (BF < 1).

### A look into the practice effect (S3 vs. SC)

Although it was not pre-registered, it was later determined that a comparison between S3 and SC was necessary to understand possible effects of practice. Results showed evidence supporting no difference (BF < 1) between these sessions for all of the variables of interest, including that of Anxiety, Depression, Loneliness, and Cognitive score (Supplementary Table 8). Bayes factor was slightly above one but less than 1.1 for Confinement as well as the interval reproduced in the paced finger-tapping task, which suggested that the available evidence is inconclusive and does not support either the presence or absence of a difference.

## Discussions

Our study aimed to investigate the effect of restricted movements during the COVID-19 pandemic on temporal cognition among the Japanese population. To achieve this, we utilized longitudinal, within subject, and between subject data collected remotely and stored in the Blursday database^23^. Given that in Japan, governmental measures were limited and abiding to these were voluntary, it was unknown whether there would be any changes in our temporal cognition.

During the first wave of the COVID-19 pandemic, there were noticeable changes in temporal cognition in various countries, including the United Kingdom^8^, Germany^18^, Italy, and France^12^. These countries implemented stringent measures, as indicated by a stringency index of over 75 out of 100 (the highest level of strictness) during their initial lockdowns according to data from Our World in Data^58^. In contrast, Japan’s stringency index only averaged 45.1 during the first confinement period, and since people had the freedom to go out, some restaurants also chose to stay open. Although Japan’s governmental measures to slow down the spread of COVID-19 was not strictly enforced, being more of recommendations on how to stay safe during the pandemic, there were observable changes in people’s mobility patterns. Google Mobility data indicated a reduction in mobility at transit stations (Supplementary Fig. 1) and workplaces, while there was an increased movement within residential areas in Japan^35^. Our main focus was therefore to explore whether and how the ability to process temporal information was affected during the pandemic and disambiguate whether and how these changes resulted from the change in mobility, psychological state, and/or cognitive ability in the Japanese population. Unlike in the previous studies looking into how the confinement period may have affected the perception of the passage of time^8,10,17^, here we focused on the production and reproduction of rhythmic temporal intervals by asking participants to perform both the spontaneous finger-tapping task, and a paced finger-tapping task.

One of the main findings of this exploratory study was that as confinement was being relaxed, the speed of the tapping in the spontaneous tapping task (produced interval) and the continuation phase of the paced finger-tapping task (reproduced interval) changed. In the spontaneous finger-tapping task, people tapped at an average pace similar to Hammerschmidt and colleagues’ study^59^, whose mean and median were around 700 ms. When looking into how the speed of the tapping changed during the pandemic in relation to other variables, the change in the mobility of people in transit appeared to be correlated. These results indicated that as people started going out more, the longer their tapping interval got; in other words, the slower they tapped. Ingram and colleagues’^14^ also found this to be the case in their production task of single intervals. In their study, they found that participants underestimated intervals during Week 1 of confinement and overestimated their intervals at Week 13 when restrictions were being lifted and thus concluded that as people felt more relaxed, the speed of the internal clock slowed down. However, the main analysis in our study using model comparison revealed that rather than the direct influence of confinement, it was the interaction of age and the confinement measure that changed how the internal clock paced (Simpson’s Paradox); in fact, for those in the later stage of adulthood, the clock paced at a faster rate (as will be discussed later).

In the paced finger-tapping task, all participants, irrespective of age, seemed to pace at a faster speed as restrictions were eased. While participants tapped at around 985 ms intervals when reproducing one-second intervals under confinement, their reproduced intervals shortened further by approximately 10 ms three months after the confinement was lifted. Though these results may seem contradictory to that of the spontaneous finger-tapping task, and function as additional evidence to show how these two tasks are performed using different mechanisms^26–30^, we can also say that as people regained their normal life style, the more their tapping rate drifted away from the one-second interval toward their comfortable tapping rate (inter-tap interval around 500–600 ms). This interpretation can also explain why accuracy in the synchronization phase was higher when participants had reduced mobility as opposed to when life resumed to normal. Thus, mixed results between the two temporal tasks may be therefore explained by the differences in the tasks as well as other factors involved in the cultural differences of confinement and further investigation may be conducted to clarify the reasoning behind these differences.

One factor that was clearly associated with the intervals produced and reproduced during confinement, was that of cognitive functions, specifically the working memory load as measured by the Three-back task. Consistent with previous studies on COVID-19 confinement^14^, our findings revealed lower performance in cognitive tests during confinement. Although not included in the top two models of the spontaneous finger tapping task (but still relatively high in the ranking of the best models—Supplementary Table 4), the produced interval was associated with the change in cognitive scores. Since cognitive scores can be used to explain the slowing of the tapping rate^60^, these correlations can provide an explanation for why the data collected during the pandemic shows people tapping at a much slower pace than the typical tapping rate of 2 Hz^61^. On the other hand, the change in both the movement of the people and cognitive ability was a good predictor in the paced finger-tapping task, in both their ability to synchronize to the external stimulus and the length of the reproduced interval. Specifically, participants exhibited the highest accuracy in the synchronization phase and a closest reproduction of one-second interval in the continuation phase when cognitive scores were highest. The fact that cognitive ability was a good predictor of the performance of the paced finger tapping task but maybe to a lesser extent in the spontaneous finger tapping task makes sense given that having to adjust the individual’s spontaneous motor tempo to a slower tempo like that of one second is cognitively demanding^29^ and thus, more likely to be affected by confinement and the cognitive ability of the participant. Hence, it seems as if both change in confinement and change in cognitive ability impacted in one way or another the temporal cognition of the participants taking part in the longitudinal study.

Another factor affecting the temporal performance in both the spontaneous finger-tapping task and the continuation phase of the paced-finger tapping task is age. More concretely, different trends for how the pandemic affected the behavioral performance was observed for different age groups. Age differences were expected since it is well known that adults with older age perform differently in many temporal tasks compared to their younger cohorts^30,61–66^ in addition to the fact that the pandemic was lived differently for those with increased age given that multiple studies show young adults suffering more in terms of mental health from the pandemic than their older cohorts^21,32–34,67–69^. Though it is well documented that older adults tap at a slower rate than younger adults^59,61^, in this concrete study, we found that in general, adults in the later stage of adulthood were tapping at a faster rate than those in the early stage of adulthood. Though at first, this may sound contradictory, it is not. Our study found that the spontaneous motor tempo of individuals varied in different directions according to age during the pandemic. While those in their 20 s and 30 s produced longer intervals as life resumed back to normal, the adults in their 50 s and 60 s produced shorter intervals under the same conditions. Finding individual differences in terms of temporal cognition during the COVID-19 pandemic is not new, given similar results have been found^8,18^, with age being associated to how we perceive the passage of time during the pandemic^8,12^. Thus, our results showed that the difference in the intervals produced in age can be explained by the varying effects of confinement for the different ages.

As for the paced finger-tapping task, a previous study by Vanneste and colleagues^61^ found no difference in the synchronization ability between older and younger adults. However, our study revealed that adults in the later stage of adulthood were better synchronized to the visual stimulus than those in the earlier stage of adulthood. This finding may be attributed to the more drastic psychological and cognitive changes experienced by younger people during the pandemic^9,21,32,33,67–69^. While indeed, cognitive ability appeared to be a factor that can predict the quality of synchronization, we did not observe any age-related variability in the psychological factors in the top models predicting the ability to synchronize to the external stimuli. Moreover, our data indicated that while adults in their 20 s and 30 s remained consistent in their ability to synchronize with visual stimuli, those in their 50 s and 60 s demonstrated improvement in their synchronization skills. While the concrete reasons behind why this could be the case are not known, it is possible that the ability to synchronize to the beat was altered in the participants in the early stage of adulthood as a result of the pandemic and this effect lingered on even after three months, whereas the adults in the late stage of adulthood improved over time. Hence, our results indicated that the ability to synchronize with a visual stimulus was not only dependent on the confinement but also by the age of the participant and their own unique experience of the pandemic.

In terms of the reproduced intervals, our results showed that the participants in the early stage of adulthood were better able to reproduce the one-second interval than the later stage of adulthood, who tended to under-reproduce the interval. This under-reproduction of the interval could be indicative of the faster internal clock speed of older adults^64,65^ or reflective of the change in internal clock speed with confinement as observed in the spontaneous finger-tapping task where the adults with increased age increasingly got faster at tapping. In fact, results also show that as people started to get used to the new normal, participants especially in their 50 s and 60 s tended to underestimate the one-second duration in the paced finger-tapping task as well. As mentioned earlier, however, these behavioral results may also indicate that as people started regaining their normal life, they were increasingly drifting towards tapping at their comfortable pace. In fact, it may be that with less restrictions, people had more things in their mind to which their attention focused on while doing the tapping task, making the reproduction of the one-second interval harder to maintain. Hence, it seems as if during the pandemic, there were differential effects of lockdown in terms of age and that the older adults seemed to increasingly under-reproduce the interval as life resumed to normal compared to the younger adults.

To our surprise, however, none of the psychological variables formed part of the predictors of the top two models for the produced duration of the spontaneous finger-tapping task nor the asynchronicity or reproduced duration of the paced finger-tapping task. Given the decrease in depression and increase in loneliness levels observed during the months of the pandemic, we had expected to see these psychological factors form part of the predictors in our models. However, this was not the case and there could be numerous reasons for this. First, it could be that the questionnaires given to the participants were more oriented toward the clinical population than the general public, hence, they could not pick up the minuscule changes in our psychological state. Yet, this seems unlikely given that differences in depression were observed between S1 and S3, as well as S1 and SC. Second, it could be that the mechanisms underlying perception, production and synchronization to one-second intervals are truly not impacted by the psychological variables. Indeed, in Mioni and colleagues’ study^16^, it seems as though the effects of depression and anxiety are most noticeable in the 500 ms duration rather than the longer ones. Thus, it is possible that psychological states did not affect the processing of 1000 ms intervals. As for the case of the spontaneous finger-tapping task, Hammerschmidt and colleagues^59^ found that when comparing the arousal level of different groups of participants with slow and fast spontaneous tapping speed, arousal levels did not matter, yet, when looking at a whole, those with higher arousal levels had faster tapping speed. Thus, in this study, it still remains unclear whether psychological variables or arousal level contributed to the changes in temporal cognition during the pandemic.

Along these lines, the third reason, and probably the most important one to note is that our statistical approach to conduct model comparisons is known to penalize complex models. This means that even if both psychological and cognitive variables are strong predictors, the model comparisons approach will favor the simpler model, which includes only one of these variables. In specific to our data, we can observe a relationship between depression and cognitive score, as well as a relationship between confinement and depression as well as loneliness. It is possible that the model selected the variable of cognitive ability as a better predictor because it is more directly tied to the temporal performance compared to depression. Though both these relationships between cognitive score and temporal performance^70,71^, as well as depression and temporal performance^72^ have been shown previously, those with depression have lower levels of cognitive abilities^73^ making it seem like those with increased levels of depression had a greater deficit in cognitive ability and therefore, were more likely to underperform in the paced-finger-tapping task. A similar reasoning would also be applied to why confinement appeared as a predictor in the top two models of temporal cognition in the paced finger-tapping task rather than depression and loneliness. Moreover, we cannot eliminate the predictor of psychological variables given that we did not statistically confirm that our top two models were significantly better than the rest. In fact, there could be a couple of models that include the psychological variable that could perform equally well as the top two models. Hence, though our results with the top two models did not point to any psychological variable being a predictor of the performance in the temporal tasks, by no means does this rule out the possibility of the psychological state affecting temporal performance but rather, it could signal that the effect of psychological state could be an indirect cause of the changes in temporal performance.

Lastly, there were a few limitations to this study. First, given this was an online study, data was strictly filtered for each of these tasks, making the participant count smaller than ideal, leading to the uncertainty presented in our model comparison results. Yet, since the Bayesian statistical approach was used, we report our uncertainty and trends rather than binary significance results. Moreover, unlike the other countries included in the Blursday Database^23^, participants from Japan were given monetary rewards to ensure that those who participated in our study would perform at their best, would participate in all sessions, and would not be excluded from the data analysis. Second, the measure used for the Confinement level was not a participant-specific measure, rather, it was a confinement measure at a societal level. Although at the point of data collection a subjective measure of how much time the participant spent outside while they participated in the task compared to how much they went outside pre-pandemic was obtained, the more objective measure obtained by the Google Mobility report was chosen for two reasons: (1) the measure of the frequency of outings is very tied with the life style and work of the participant—for example, even if the participant does not want to go out, due to fears of the pandemic, if her work does not allow teleworking, he or she would spend more time outside the house than what they wish to, and (2) fact that the subjective measure relies on how much the participant went out this year compared to last year, requires an estimation of time that may not be accurate, (3) though Google Mobility report is not measured at an individual level, it not only reflects how much people go out, rather it can be indicative of how much people are willing to go out. Even if governmental measures for the response to the pandemic were not strictly enforced, there was clear evidence of people’s mobility being more restricted in S1 compared to S3 and SC, showing that people were more likely to follow the societal norm. Last, the models studied here are only a few of all the possibilities. Though we managed to compare over 40 models for each of the three temporal variables, this was nowhere close to the number of all the possibilities. There are factors such as that of perceived control over aversive stimuli^74^ and boredom^12,13^ that could also explain the behavioral changes found in confinement but were not tested simply due to the fact that we could not account for all possible variables in this exploratory study. Likewise, other well-established aspects of time processing, namely the decomposition of the variance into central and motor components (i.e. Wing & Kristofferson’s two-level model^75^) were not taken into consideration despite being potentially related to some of our factors^76^. In addition, it may have been more appropriate to use a non-linear model since the variables measured do not change linearly, rather at some point, it can plateau. However, given that we only had three data points in the longitudinal data, using quadratic equations was not possible. All this in mind, however, our results can add to the understanding of how the pandemic was experienced in Japan and the trend in the changes in the mental state and cognitive ability, as well as those produced temporal intervals in Japan.

In conclusion, in this exploratory study, we analyzed both longitudinal and cross-sectional data in order to get an idea of how our spontaneous motor tempo and our reproduction of one second, along with our cognitive abilities as well as the psychological state of anxiety, depression and loneliness levels may have been impacted by the pandemic.

## Supplementary Information

Supplementary Information.

## Data Availability

All data used for this project is available on the Blursday Database^23^ and the analysis scripts, and all modeling results are available upon request. Please contact the corresponding author, EMGH, for the necessary materials.
