# Supplementary material for: Longitudinal analysis of social isolation effects on finger tapping in the Blursday database
Source: Sci Rep. 2023 Jul 12;13:11277. doi: 10.1038/s41598-023-38488-w (PMC10338501; doi:10.1038/s41598-023-38488-w)
Supplement: Supplementary file 1 — Supplementary Information. [file 41598_2023_38488_MOESM1_ESM.docx]

Supplementary Figure 1: The movement of people in transit stations in Tokyo relative to the movement of people during January and February of 2020 according to the Google Mobility Report (Google LLC, n.d.). While the grey lines depict the movement recorded on that day, the dark black lines illustrate the seven-day rolling average. The shaded rectangles illustrate the period in which the data was collected (S1: Apr. 20^th^, 2020 – May 26^th^, 2020; S2: Jun. 8^th^, 2020 – Jul. 7^th^, 2020; S3: Sept. 23^rd^, 2020 – Oct. 23^rd^, 2020; SC: Jul. 1^st^, 2021 – Jul. 21^st^, 2021) and the color bar in the x-axis depicts the dates of the State of Emergency (SOE) and weakened SOE (light SOE) in Tokyo (SOE 1: Apr.7^th^, 2020 – May 25^th^, 2020 ; SOE 2: Jan. 8^th^, 2021 – Mar. 21^st^, 2021; lightSOE 1: Apr. 12^th^, 2021 – Apr. 24^th^, 2021; SOE 4: Apr. 25^th^, 2021 – June 20^th^, 2021; lightSOE: June 21^st^, 2021 – Jul. 10^th^, 2021; SOE 4: Jul 11^th^, 2021 – Sept. 30^th^, 2021).


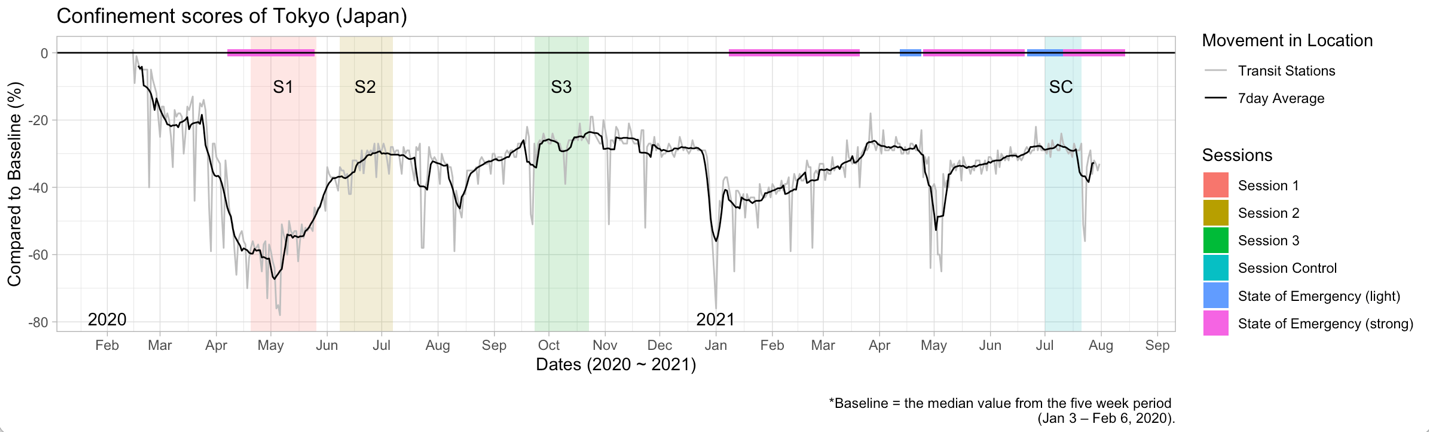


**Supplementary Figure 2: The age distribution of the participants who were included into the study.** Note that when recruiting participants, an equal number of individuals were initially recruited for each age group at S1 (for the longitudinal study) and at SC (new/naïve participants). However, it is important to acknowledge that participants had the freedom to drop out at any point during the study, and consequently, some individuals chose to withdraw during the middle of session 1. As a result, the count of participants in their sixties at the beginning of S1 is significantly lower compared to the other age groups, due to these dropouts.


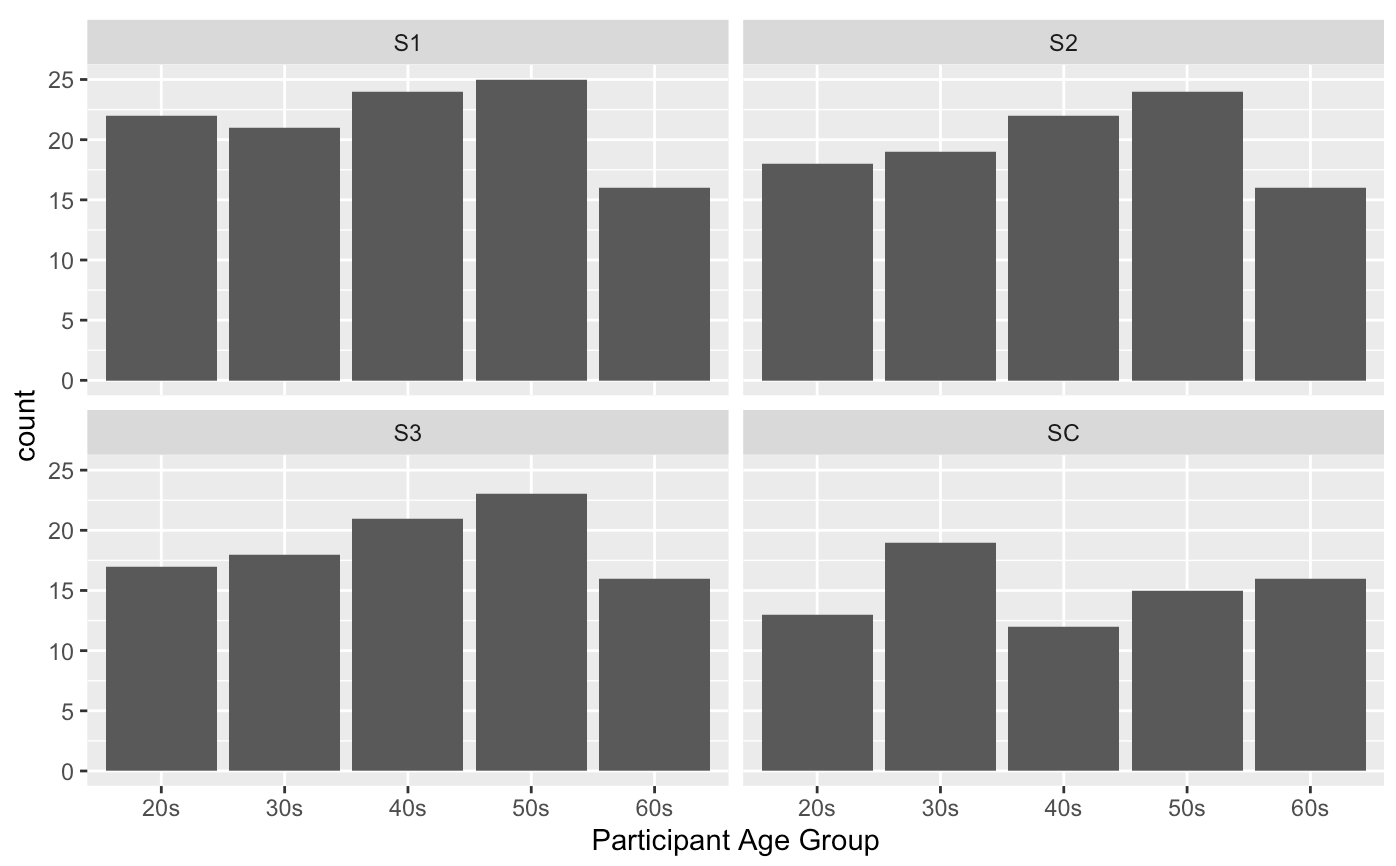


Supplementary Material 1: Exclusion of the paced finger-tapping task:

**1. Synchronization phase**

First, we excluded trials with a number of either bips or taps greater than 100 in the synchronization phase since it meant that there was either a software glitch (number of bips greater than the intended 60) or the participant tapped much more often than once per bip, respectively.

After this, trials were discarded according to the following criteria in this order:

- **Drifting subjects.** We computed an “agnostic” measure of the phase difference between the sequence of responses and the sequence of stimuli for every trial and estimated its slope when plotted as a function of time (using circular statistics; we implemented the proposal of (Kempter et al., 2012).Any trial with an absolute value of the phase slope greater than pi/60s (equivalent to drifting a whole circle from beginning to end of the trial) was classified as an outlier and removed.
- **Confused instructions.** We computed the mean phase for every trial (circular mean from the R package circular, Lund et al., 2022) and detected outliers by using the standard outlier definition of the boxplot.stats function. The discarded trials had a mean phase either close to +/-pi during InSync or close to zero during OutSync, meaning that participants tapped out-of-phase when instructed to do it in-phase and vice versa.
- **Exceedingly variable trials.** We computed the phase-locking value (Lachaux et al., 1999, adapted to paced finger tapping) for every trial and discarded the low-end outliers of the phase-locking value distribution by using the boxplot.stats function. We also discarded the high-end outliers of the distribution of circular standard deviation (from the R package circular) of the phase.
- **Reaction instead of synchronization.** To detect trials where subjects were reacting to every stimulus instead of synchronizing to the stimuli sequence we estimated a clustering of the trial data in a three-dimensional space (because no single variable could clearly cut them off). The chosen variables were the trial median asynchrony, trial median absolute deviation (MAD) of asynchronies, and the number of large asynchronies of the trial (i.e. the number of responses with asynchrony greater than 200 ms in InSync and lesser than -200 ms in OutSync). The clustering was estimated via the Mclust function in the R package mclust (with default algorithm expectation-maximization, Fraley et al., 2022). In this three-dimensional space the trials belonging to the only cluster with consistently high trial median asynchrony, low trial MAD of asynchronies, and high number of large asynchronies were discarded as outliers (the cluster was easily spotted by a high value of the third variable).
- **Too few surviving responses.** Any trial with less than 30 valid responses (half the number of stimuli) was discarded.

**2. Continuation phase**

The outlier criteria for the continuation phase were very similar to the criteria for the free-tapping task, and were applied after the removal of the synchronization phase outliers and after excluding continuation phases with more than 300 taps (9.6% of the taps). First, from every trial from every subject we discarded single ITIs with a duration smaller than 1 ms and larger than 5 s. Second, we discarded whole trials if they had at least 30 ITIs with a duration of less than 50 ms (key continuously pressed down). Finally, we discarded whole trials if their median ITI was detected as an outlier by the function outlier_mad described above with a threshold of three.

**Supplementary Table 1:** Models included in the analysis for predictors of temporal tasks during COVID-19 (Part 4, Experiment 4.1 and Experiment 4.2). The variable TempV can take the place of the produced interval in the spontaneous finger-tapping task (Experiment 4.1), as well as the asynchrony measure and the reproduced interval in the paced finger-tapping task (Experiment 4.2).

|  | Model Description |
| --- | --- |
| Model Confinement | TempV ~ 1 + Confinement |
| Model Age | TempV ~ 1 + Age |
| Model Age:Confinement | TempV ~ 1 + Age:Confinement |
| Model Basic | TempV ~ 1 + Confinement + Age + Age:Confinement + (1 \| pid) |
| Model Cog | TempV ~ 1 + Confinement + Age + Age:Confinement + Cognitive + (1 + Cognitive\|pid) |
| Model Psych | TempV ~ 1 + Confinement + Age + Age:Confinement + Anxiety + Depression + Loneliness + (1 + Anxiety + Depression + Loneliness\|pid) |
| Model Anxiety | TempV ~ 1 + Confinement + Age + Age:Confinement + Anxiety + (1 + Anxiety\|pid) |
| Model Depression | TempV ~ 1 + Confinement + Age + Age:Confinement + Depression + (1 + Depression\|pid) |
| Model Loneliness | TempV ~ 1 + Confinement + Age + Age:Confinement + Loneliness + (1 + Loneliness\|pid) |
| Model Cog Interaction | TempV ~ 1 + Confinement + Age + Age:Confinement + Age:Cognitive + Confinement:Cognitive + (1 + Cognitive\|pid) |
| Model Psych Interaction | TempV ~ 1 + Confinement + Age + Age:Confinement + Anxiety + Depression + Loneliness + Age:Anxiety + Age:Depression + Age:Loneliness + Confinement:Anxiety + Confinement:Depression + Confinement + Loneliness + (1 + Anxiety + Depression + Loneliness\|pid) |
| Model Anxiety Interaction | TempV ~ 1 + Confinement + Age + Age:Confinement + Anxiety + Age:Anxiety + Confinement:Anxiety + (1 + Anxiety\|pid) |
| Model Depression Interaction | TempV ~ 1 + Confinement + Age + Age:Confinement + Depression + Age:Depression + Confinement:Depression + (1 + Depression\|pid) |
| Model Loneliness Interaction | TempV ~ 1 + Confinement + Age + Age:Confinement + Loneliness + Age:Loneliness + Confinement:Loneliness + (1 + Loneliness\|pid) |
| Model Age Interaction | TempV ~ 1 + Confinement + Age + Age:Confinement + Cognitive + Anxiety + Depression + Loneliness + Confinement:Cognitive + Confinement:Anxiety + Confinement:Depression + Confinement:Loneliness + (1 + Cognitive + Anxiety + Depression + Loneliness\|pid) |
| Model Confinement Interaction | TempV ~ 1 + Confinement + Age + Age:Confinement + Cognitive + Anxiety + Depression + Loneliness + Age:Cognitive + Age:Anxiety + Age:Depression + Age:Loneliness + (1 + Cognitive + Anxiety + Depression + Loneliness\|pid) |
| Model Basic_noConf | TempV ~ 1 + Age + Age:Confinement + (1 \| pid) |
| Model Cog_noConf | TempV ~ 1 + Age + Age:Confinement + Cognitive + (1 + Cognitive\|pid) |
| Model Psych_noConf | TempV ~ 1 + Age + Age:Confinement + Anxiety + Depression + Loneliness + (1 + Anxiety + Depression + Loneliness\|pid) |
| Model Anxiety_noConf | TempV ~ 1 + Age + Age:Confinement + Anxiety + (1 + Anxiety\|pid) |
| Model Depression_noConf | TempV ~ 1 + Age + Age:Confinement + Depression + (1 + Depression\|pid) |
| Model Loneliness_noConf | TempV ~ 1 + Age + Age:Confinement + Loneliness + (1 + Loneliness\|pid) |
| Model Cog Interaction_noConf | TempV ~ 1 + Age + Age:Confinement + Age:Cognitive + Confinement:Cognitive + (1 + Cognitive\|pid) |
| Model Psych Interaction_noConf | TempV ~ 1 + Age + Age:Confinement + Anxiety + Depression + Loneliness + Age:Anxiety + Age:Depression + Age:Loneliness + Confinement:Anxiety + Confinement:Depression + Confinement + Loneliness + (1 + Anxiety + Depression + Loneliness\|pid) |
| Model Anxiety Interaction_noConf | TempV ~ 1 + Age + Age:Confinement + Anxiety + Age:Anxiety + Confinement:Anxiety + (1 + Anxiety\|pid) |
| Model Depression Interaction_noConf | TempV ~ 1 + Age + Age:Confinement + Depression + Age:Depression + Confinement:Depression + (1 + Depression\|pid) |
| Model Loneliness Interaction_noConf | TempV ~ 1 + Age + Age:Confinement + Loneliness + Age:Loneliness + Confinement:Loneliness + (1 + Loneliness\|pid) |
| Model Age Interaction_noConf | TempV ~ 1 + Age + Age:Confinement + Cognitive + Anxiety + Depression + Loneliness + Confinement:Cognitive + Confinement:Anxiety + Confinement:Depression + Confinement:Loneliness + (1 + Cognitive + Anxiety + Depression + Loneliness\|pid) |
| Model Confinement Interaction_noConf | TempV ~ 1 + Age + Age:Confinement + Cognitive + Anxiety + Depression + Loneliness + Age:Cognitive + Age:Anxiety + Age:Depression + Age:Loneliness + (1 + Cognitive + Anxiety + Depression + Loneliness\|pid) |
| Model Basic_noAge:Conf | TempV ~ 1 + Confinement + Age + (1 \| pid) |
| Model Cog_noAge:Conf | TempV ~ 1 + Confinement + Age + Cognitive + (1 + Cognitive\|pid) |
| Model Psych_noAge:Conf | TempV ~ 1 + Confinement + Age + Anxiety + Depression + Loneliness + (1 + Anxiety + Depression + Loneliness\|pid) |
| Model Anxiety_noAge:Conf | TempV ~ 1 + Confinement + Age + Anxiety + (1 + Anxiety\|pid) |
| Model Depression_noAge:Conf | TempV ~ 1 + Confinement + Age + Depression + (1 + Depression\|pid) |
| Model Loneliness_noAge:Conf | TempV ~ 1 + Confinement + Age + Loneliness + (1 + Loneliness\|pid) |
| Model Cog Interaction_noAge:Conf | TempV ~ 1 + Confinement + Age + Age:Cognitive + Confinement:Cognitive + (1 + Cognitive\|pid) |
| Model Psych Interaction_noAge:Conf | TempV ~ 1 + Confinement + Age + Anxiety + Depression + Loneliness + Age:Anxiety + Age:Depression + Age:Loneliness + Confinement:Anxiety + Confinement:Depression + Confinement + Loneliness + (1 + Anxiety + Depression + Loneliness\|pid) |
| Model Anxiety Interaction_noAge:Conf | TempV ~ 1 + Confinement + Age + Anxiety + Age:Anxiety + Confinement:Anxiety + (1 + Anxiety\|pid) |
| Model Depression Interaction_noAge:Conf | TempV ~ 1 + Confinement + Age + Depression + Age:Depression + Confinement:Depression + (1 + Depression\|pid) |
| Model Loneliness Interaction_noAge:Conf | TempV ~ 1 + Confinement + Age + Loneliness + Age:Loneliness + Confinement:Loneliness + (1 + Loneliness\|pid) |
| Model Age Interaction_noAge:Conf | TempV ~ 1 + Confinement + Age + Cognitive + Anxiety + Depression + Loneliness + Confinement:Cognitive + Confinement:Anxiety + Confinement:Depression + Confinement:Loneliness + (1 + Cognitive + Anxiety + Depression + Loneliness\|pid) |
| Model Confinement Interaction_noAge:Conf | TempV ~ 1 + Confinement + Age + Cognitive + Anxiety + Depression + Loneliness + Age:Cognitive + Age:Anxiety + Age:Depression + Age:Loneliness + (1 + Cognitive + Anxiety + Depression + Loneliness\|pid) |

**Supplementary Table 2:** Bayesian Pearson Correlation results between the change in psychological, cognitive and confinement measures as well as the change in the produced duration (Spontaneous ITI) in the spontaneous finger-tapping task, the asynchrony measure (Paced Asyn), and the reproduced duration (Paced ITI) in the paced finger-tapping task.

|  | |  | | **Age** | | **Change in Anxiety** | | **Change in Depression** | | **Change in Loneliness** | | **Change in Cognitive** | | **Change in Confinement** | | **Change in Spontaneous ITI** | | **Change in Paced Asyn** | | **Change in Paced ITI** | |
| --- | --- | --- | --- | --- | --- | --- | --- | --- | --- | --- | --- | --- | --- | --- | --- | --- | --- | --- | --- | --- | --- |
| Age |  | Pearson's r |  | — |  |  |  |  |  |  |  |  |  |  |  |  |  |  |  |  |  |
|  |  | BF₁₀ |  | — |  |  |  |  |  |  |  |  |  |  |  |  |  |  |  |  |  |
| Change in Anxiety |  | Pearson's r |  | 0.043 |  | — |  |  |  |  |  |  |  |  |  |  |  |  |  |  |  |
|  |  | BF₁₀ |  | 0.097 |  | — |  |  |  |  |  |  |  |  |  |  |  |  |  |  |  |
| Change in Depression |  | Pearson's r |  | 0.052 |  | 0.311 | *** | — |  |  |  |  |  |  |  |  |  |  |  |  |  |
|  |  | BF₁₀ |  | 0.109 |  | 105986.561 |  | — |  |  |  |  |  |  |  |  |  |  |  |  |  |
| Change in Loneliness |  | Pearson's r |  | 0.085 |  | 0.143 |  | 0.024 |  | — |  |  |  |  |  |  |  |  |  |  |  |
|  |  | BF₁₀ |  | 0.207 |  | 1.351 |  | 0.081 |  | — |  |  |  |  |  |  |  |  |  |  |  |
| Change in Cognitive |  | Pearson's r |  | -0.018 |  | 0.104 |  | -0.163 |  | 0.038 |  | — |  |  |  |  |  |  |  |  |  |
|  |  | BF₁₀ |  | 0.079 |  | 0.332 |  | 3.016 |  | 0.092 |  | — |  |  |  |  |  |  |  |  |  |
| Change in Confinement |  | Pearson's r |  | 0.023 |  | 0.002 |  | -0.229 | *** | 0.149 |  | 0.425 | *** | — |  |  |  |  |  |  |  |
|  |  | BF₁₀ |  | 0.080 |  | 0.074 |  | 135.622 |  | 1.728 |  | 5.205e +10 |  | — |  |  |  |  |  |  |  |
| Change in Spontaneous ITI |  | Pearson's r |  | -0.038 |  | -0.026 |  | -0.040 |  | 0.042 |  | 0.184 |  | 0.240 | *** | — |  |  |  |  |  |
|  |  | BF₁₀ |  | 0.092 |  | 0.083 |  | 0.094 |  | 0.096 |  | 7.988 |  | 241.383 |  | — |  |  |  |  |  |
| Change in Paced Asyn |  | Pearson's r |  | -0.172 |  | -0.055 |  | -0.004 |  | 0.042 |  | -0.010 |  | 0.048 |  | 0.121 |  | — |  |  |  |
|  |  | BF₁₀ |  | 0.947 |  | 0.126 |  | 0.101 |  | 0.115 |  | 0.103 |  | 0.120 |  | 0.305 |  | — |  |  |  |
| Change in Paced ITI |  | Pearson's r |  | -0.023 |  | 0.048 |  | 0.070 |  | 0.129 |  | 0.070 |  | 0.012 |  | 0.098 |  | 0.023 |  | — |  |
|  |  | BF₁₀ |  | 0.087 |  | 0.107 |  | 0.143 |  | 0.563 |  | 0.143 |  | 0.083 |  | 0.248 |  | 0.111 |  | — |  |
|  | | | | | | | | | | | | | | | | | | | | | |
| * BF₁₀ > 10, ** BF₁₀ > 30, *** BF₁₀ > 100 | | | | | | | | | | | | | | | | | | | | | |

**Supplementary Table 3:** Bayesian Independent T-test samples comparing the variables of interest (Anxiety, Depression, Loneliness, Cognitive, and Confinement score, as well as the temporal performance) between S1 and S3. Temporal performance is denoted as “Spontaneous ITI” for produced duration in the spontaneous finger-tapping task, “Paced Asyn” for the asynchrony measure of the paced finger-tapping task and “Paced ITI” for the reproduced duration in the paced finger-tapping task.

| **Bayesian Independent Samples T-Test** | | | | | |
| --- | --- | --- | --- | --- | --- |
|  | | **BF₁₀** | | **error %** | |
| Anxiety |  | 0.153 |  | 8.393e  -7 |  |
| Depression |  | 1.416 |  | 1.091e  -6 |  |
| Loneliness |  | 0.264 |  | 1.370e  -6 |  |
| Cognitive |  | 399.727 |  | 1.178e  -8 |  |
| Confinement |  | 2.135e +98 |  | 4.958e -101 |  |
| Spontaneous_ITI |  | 1.190 |  | 1.574e  -6 |  |
| Paced_Asyn |  | 0.195 |  | 0.009 |  |
| Paced_ITI |  | 0.189 |  | 1.274e  -5 |  |
|  | | | | | |

| **Descriptives** | | | | | | | | | | | | | | | |
| --- | --- | --- | --- | --- | --- | --- | --- | --- | --- | --- | --- | --- | --- | --- | --- |
|  | | | | | | | | | | | **95% Credible Interval** | | | | |
|  | | **Group** | | **N** | | **Mean** | | **SD** | | **SE** | **Lower** | | | **Upper** | |
| Anxiety |  | S1 |  | 108.000 |  | 5.352 |  | 3.347 |  | 0.322 |  | 4.713 |  | 5.990 |  |
|  |  | S3 |  | 95.000 |  | 5.326 |  | 3.419 |  | 0.351 |  | 4.630 |  | 6.023 |  |
| Depression |  | S1 |  | 108.000 |  | 9.019 |  | 3.724 |  | 0.358 |  | 8.308 |  | 9.729 |  |
|  |  | S3 |  | 95.000 |  | 7.863 |  | 3.791 |  | 0.389 |  | 7.091 |  | 8.636 |  |
| Loneliness |  | S1 |  | 108.000 |  | 19.157 |  | 11.822 |  | 1.138 |  | 16.902 |  | 21.413 |  |
|  |  | S3 |  | 95.000 |  | 21.053 |  | 13.188 |  | 1.353 |  | 18.366 |  | 23.739 |  |
| Cognitive |  | S1 |  | 107.000 |  | 76.367 |  | 7.901 |  | 0.764 |  | 74.853 |  | 77.882 |  |
|  |  | S3 |  | 93.000 |  | 81.043 |  | 7.959 |  | 0.825 |  | 79.404 |  | 82.683 |  |
| Confinement |  | S1 |  | 108.000 |  | -58.042 |  | 6.425 |  | 0.618 |  | -59.268 |  | -56.817 |  |
|  |  | S3 |  | 95.000 |  | -27.746 |  | 2.803 |  | 0.288 |  | -28.317 |  | -27.175 |  |
| Spontaneous_ITI |  | S1 |  | 106.000 |  | 676.347 |  | 309.705 |  | 30.081 |  | 616.701 |  | 735.992 |  |
|  |  | S3 |  | 94.000 |  | 764.215 |  | 279.212 |  | 28.799 |  | 707.027 |  | 821.403 |  |
| Paced_Asyn |  | S1 |  | 58.000 |  | 26.626 |  | 73.019 |  | 9.588 |  | 7.427 |  | 45.826 |  |
|  |  | S3 |  | 61.000 |  | 26.448 |  | 59.479 |  | 7.615 |  | 11.215 |  | 41.682 |  |
| Paced_ITI |  | S1 |  | 89.000 |  | 977.793 |  | 79.048 |  | 8.379 |  | 961.141 |  | 994.444 |  |
|  |  | S3 |  | 83.000 |  | 971.729 |  | 67.827 |  | 7.445 |  | 956.918 |  | 986.539 |  |
|  | | | | | | | | | | | | | | | |

**Supplementary Table 4:** Model comparison using WAIC and global WAIC for the gaussian model family as implemented in the glmmstan in the R package for the produced interval in the spontaneous finger-tapping task. For all models, we assumed that the intercept of the temporal measures was dependent on each participant. When the models included psychological or cognitive variables, we assumed that intercepts and slopes for these scores for each participant were random effects.

|  | WAIC | | | WAICg | | |
| --- | --- | --- | --- | --- | --- | --- |
|  | Model | Model_noConf | Model_noConf:Age | Model | Model_noConf | Model_noConf:Age |
| **Models with no Cog or Psych effect** | | | | | | |
| Model Confinement | 3673.187 | NA | NA | 4054.449 | NA | NA |
| Model Age | 4012.100 | NA | NA | 4376.816 | NA | NA |
| Model Age:Confinement | 3695.134 | NA | NA | 4034.802 | NA | NA |
| Model with All above | 3675.716 | 3695.340 | 3673.440 | 4064.578 | 4036.394 | 4062.685 |
| **Models with Cog or Psych effect** | | | | | | |
| Model Cog | 3627.076 | 3631.846 | 3625.960 | 4053.480 | 4044.725 | 4049.095 |
| Model Psych | 3676.846 | 3695.761 | 3674.600 | 4067.343 | 4039.113 | 4065.566 |
| Model Anxiety | 3658.918 | 3695.218 | 3675.395 | 4057.245 | 4039.613 | 4065.292 |
| Model Depression | 3671.073 | 3688.735 | 3669.355 | 4074.346 | 4051.349 | 4073.047 |
| Model Loneliness | 3674.711 | 3691.862 | 3675.118 | 4134.902 | 4102.807 | 4134.011 |
| Model Cog Interaction | 3645.070 | 3652.972 | 3644.948 | 4055.500 | 4039.777 | 4054.030 |
| Model Psych Interaction | 3678.005 | 3685.229 | 3678.587 | 4171.496 | 4152.320 | 4105.933 |
| Model Anxiety Interaction | 3680.799 | 3700.657 | 3679.046 | 4071.969 | 4043.584 | 4069.043 |
| Model Depression Interaction | 3677.661 | 3692.168 | 3675.264 | 4081.782 | 4057.739 | 4081.160 |
| Model Loneliness Interaction | 3673.296 | 3682.755 | 3671.657 | 4086.433 | 4057.696 | 4087.098 |
| Model Confinement Interaction | 3630.645 | 3633.071 | 3629.346 | 4125.374 | 4123.144 | 4127.575 |
| Model Age Interaction | 3637.156 | 3645.855 | 3637.304 | 4107.635 | 4085.676 | 4103.929 |

**Supplementary Table 5:** Model comparison using WAIC and global WAIC for the gaussian model family as implemented in the glmmstan in the R package for the measure of asynchrony in the paced finger-tapping task. For all models, we assumed that the intercept of the temporal measures was dependent on each participant. When the models included psychological or cognitive variables, we assumed that intercepts and slopes for these scores for each participant were random effects.

|  | WAIC | | | WAICg | | |
| --- | --- | --- | --- | --- | --- | --- |
|  | Model | Model_noConf | Model_noConf:Age | Model | Model_noConf | Model_noConf:Age |
| **Models with no Cog or Psych effect** | | | | | | |
| Model Confinement | 1618.681 | NA | NA | 1826.527 | NA | NA |
| Model Age | 1934.023 | NA | NA | 2120.061 | NA | NA |
| Model Age:Confinement | 1616.385 | NA | NA | 1818.641 | NA | NA |
| Model with All above | 1616.040 | 1617.108 | 1619.140 | 1830.819 | 1830.342 | 1829.018 |
| **Models with Cog or Psych effect** | | | | | | |
| Model Cog | 1582.236 | 1583.923 | 1585.341 | 1742.780 | 1744.207 | 1741.754 |
| Model Psych | 1738.892 | 1741.209 | 1740.839 | 1888.870 | 1882.204 | 1886.022 |
| Model Anxiety | 1736.831 | 1737.553 | 1737.031 | 1895.822 | 1892.755 | 1890.762 |
| Model Depression | 1739.022 | 1740.852 | 1738.468 | 1886.937 | 1880.574 | 1883.453 |
| Model Loneliness | 1619.109 | 1619.773 | 1622.349 | 1852.105 | 1848.494 | 1850.078 |
| Model Cog Interaction | 1585.610 | 1587.610 | 1588.454 | 1744.263 | 1744.313 | 1746.205 |
| Model Psych Interaction | NA | 1623.865 | 1626.490 | NA | 1880.511 | 1872.289 |
| Model Anxiety Interaction | 1742.731 | 1744.537 | 1742.103 | 1888.966 | 1883.251 | 1885.024 |
| Model Depression Interaction | 1737.066 | 1740.233 | 1734.768 | 1901.831 | 1891.340 | 1899.037 |
| Model Loneliness Interaction | 1741.167 | 1742.206 | 1739.263 | 1895.614 | 1891.224 | 1893.934 |
| Model Confinement Interaction | 1590.595 | 1587.956 | 1592.896 | 1762.891 | 1754.429 | 1762.858 |
| Model Age Interaction | 1586.028 | 1588.873 | 1586.660 | 1783.495 | 1778.080 | 1779.262 |

**Supplementary Table 6:** Model comparison using WAIC and global WAIC for the gaussian model family as implemented in the glmmstan in the R package for the reproduced interval in the paced finger-tapping task. For all models, we assumed that the intercept of the temporal measures was dependent on each participant. When the models included psychological or cognitive variables, we assumed that intercepts and slopes for these scores for each participant were random effects.

|  | WAIC | | | WAICg | | |
| --- | --- | --- | --- | --- | --- | --- |
|  | Model | Model_noConf | Model_noConf:Age | Model | Model_noConf | Model_noConf:Age |
| **Models with no Cog or Psych effect** | | | | | | |
| Model Confinement | 2577.886 | NA | NA | 2658.006 | NA | NA |
| Model Age | 2882.315 | NA | NA | 2942.026 | NA | NA |
| Model Age:Confinement | 2577.814 | NA | NA | 2656.857 | NA | NA |
| Model with All above | 2579.448 | 2576.756 | 2577.092 | 2651.544 | 2651.964 | 2650.363 |
| **Models with Cog or Psych effect** | | | | | | |
| Model Cog | 2543.283 | 2540.578 | 2541.186 | 2632.642 | 2634.322 | 2631.352 |
| Model Psych | 2581.340 | 2578.489 | 2579.285 | 2654.951 | 2655.411 | 2653.686 |
| Model Anxiety | 2581.711 | 2578.767 | 2579.281 | 2656.006 | 2656.822 | 2654.747 |
| Model Depression | 2583.124 | 2580.621 | 2580.717 | 2653.107 | 2653.496 | 2652.187 |
| Model Loneliness | 2586.188 | 2583.681 | 2584.029 | 2661.819 | 2662.457 | 2660.704 |
| Model Cog Interaction | 2546.572 | 2543.164 | 2544.822 | 2637.547 | 2639.315 | 2636.261 |
| Model Psych Interaction | NA | 2584.542 | 2584.382 | NA | 2662.136 | 2660.157 |
| Model Anxiety Interaction | 2583.719 | 2581.062 | 2579.969 | 2652.069 | 2652.633 | 2650.747 |
| Model Depression Interaction | 2583.244 | 2580.726 | 2581.057 | 2656.744 | 2658.034 | 2656.008 |
| Model Loneliness Interaction | 2581.492 | 2579.130 | 2579.244 | 2654.297 | 2654.357 | 2653.236 |
| Model Confinement Interaction | 2552.449 | 2547.464 | 2549.746 | 2652.813 | 2655.601 | 2651.745 |
| Model Age Interaction | 2545.556 | 2543.286 | 2544.275 | 2641.334 | 2643.102 | 2641.430 |

**Supplementary Table 7:** Bayesian Independent T-test samples comparing the variables of interest (Anxiety, Depression, Loneliness, Cognitive, and Confinement score, as well as the temporal performance) between S1 and SC. Temporal performance is denoted as “Spontaneous ITI” for produced duration in the spontaneous finger-tapping task, “Paced Asyn” for the asynchrony measure of the paced finger-tapping task and “Paced ITI” for the reproduced duration in the paced finger-tapping task.

| **Bayesian Independent Samples T-Test** | | | | | |
| --- | --- | --- | --- | --- | --- |
|  | | **BF₁₀** | | **error %** | |
| Age |  | 0.198 |  | 1.207e  -5 |  |
| Anxiety |  | 0.168 |  | 0.046 |  |
| Depression |  | 13.022 |  | 2.161e  -7 |  |
| Loneliness |  | 0.184 |  | 1.306e  -5 |  |
| Cognitive |  | 6.749 |  | 4.827e  -7 |  |
| Confinement |  | 6.968e +85 |  | 8.118e -89 |  |
| Spontaneous_ITI |  | 0.224 |  | 1.137e  -5 |  |
| Paced_Asyn |  | 0.407 |  | 3.284e  -5 |  |
| Paced_ITI |  | 0.329 |  | 5.087e  -7 |  |
|  | | | | | |

| **Descriptives** | | | | | | | | | | | | | | | | | | |  |  |
| --- | --- | --- | --- | --- | --- | --- | --- | --- | --- | --- | --- | --- | --- | --- | --- | --- | --- | --- | --- | --- |
|  | | | | | | | | | | | | | | | **95% Credible Interval** | | | |  |  |
|  | **Group** | | | **N** | **Mean** | | | | **SD** | | **SE** | | | | **Lower** | | **Upper** | |  |  |
| Age |  | S1 |  | 108 |  | 43.389 |  | 12.571 | |  | | 1.210 | |  | | 40.991 |  | 45.787 | |  |
|  |  | SC |  | 70 |  | 44.614 |  | 13.392 | |  | | | 1.601 |  | | 41.421 |  | 47.807 | |  |
| Anxiety |  | S1 |  | 108 |  | 5.352 |  | 3.347 | |  | | | 0.322 |  | | 4.713 |  | 5.990 | |  |
|  |  | SC |  | 72 |  | 5.236 |  | 3.973 | |  | | | 0.468 |  | | 4.302 |  | 6.170 | |  |
| Depression |  | S1 |  | 108 |  | 9.019 |  | 3.724 | |  | | | 0.358 |  | | 8.308 |  | 9.729 | |  |
|  |  | SC |  | 72 |  | 7.361 |  | 3.186 | |  | | | 0.375 |  | | 6.612 |  | 8.110 | |  |
| Loneliness |  | S1 |  | 108 |  | 19.157 |  | 11.822 | |  | | | 1.138 |  | | 16.902 |  | 21.413 | |  |
|  |  | SC |  | 72 |  | 20.014 |  | 11.354 | |  | | | 1.338 |  | | 17.346 |  | 22.682 | |  |
| Cognitive |  | S1 |  | 107 |  | 76.367 |  | 7.901 | |  | | | 0.764 |  | | 74.853 |  | 77.882 | |  |
|  |  | SC |  | 73 |  | 79.828 |  | 8.145 | |  | | | 0.953 |  | | 77.928 |  | 81.729 | |  |
| Confinement |  | S1 |  | 108 |  | -58.042 |  | 6.425 | |  | | | 0.618 |  | | -59.268 |  | -56.817 | |  |
|  |  | SC |  | 73 |  | -28.397 |  | 0.602 | |  | | | 0.070 |  | | -28.538 |  | -28.257 | |  |
| Spontaneous_ITI |  | S1 |  | 106 |  | 682.855 |  | 311.434 | |  | | | 30.249 |  | | 622.877 |  | 742.834 | |  |
|  |  | SC |  | 73 |  | 722.990 |  | 343.650 | |  | | | 40.221 |  | | 642.810 |  | 803.169 | |  |
| Paced_Asyn |  | S1 |  | 58 |  | 26.626 |  | 73.019 | |  | | | 9.588 |  | | 7.427 |  | 45.826 | |  |
|  |  | SC |  | 47 |  | 3.340 |  | 119.247 | |  | | | 17.394 |  | | -31.672 |  | 38.352 | |  |
| Paced_ITI |  | S1 |  | 88 |  | 980.313 |  | 75.819 | |  | | | 8.082 |  | | 964.249 |  | 996.378 | |  |
|  |  | SC |  | 59 |  | 994.176 |  | 65.793 | |  | | | 8.566 |  | | 977.030 |  | 1011.322 | |  |
|  | | | | | | | | | | | | | | | | | | |  |  |

**Supplementary Table 8:** Bayesian Independent T-test samples comparing the variables of interest (Anxiety, Depression, Loneliness, Cognitive, and Confinement score, as well as the temporal performance) between S3 and SC. Temporal performance is denoted as “Spontaneous ITI” for produced duration in the spontaneous finger-tapping task, “Paced Asyn” for the asynchrony measure of the paced finger-tapping task and “Paced ITI” for the reproduced duration in the paced finger-tapping task.

| **Bayesian Independent Samples T-Test** | | | | | |
| --- | --- | --- | --- | --- | --- |
|  | | **BF₁₀** | | **error %** | |
| Age |  | 0.173 |  | 1.027e -5 |  |
| Anxiety |  | 0.170 |  | 1.151e -5 |  |
| Depression |  | 0.250 |  | 8.437e -6 |  |
| Loneliness |  | 0.194 |  | 1.037e -5 |  |
| Cognitive |  | 0.256 |  | 8.178e -6 |  |
| Confinement |  | 1.023 |  | 2.322e -6 |  |
| Spontaneous_ITI |  | 0.263 |  | 8.226e -6 |  |
| Paced_Asyn |  | 0.445 |  | 2.329e -5 |  |
| Paced_ITI |  | 1.056 |  | 0.009 |  |
|  | | | | | |

| **Descriptives** | | | | | | | | | | | | | | | |
| --- | --- | --- | --- | --- | --- | --- | --- | --- | --- | --- | --- | --- | --- | --- | --- |
|  | | | | | | | | | | | | **95% Credible Interval** | | | |
|  | | **Group** | | **N** | | **Mean** | | **SD** | | **SE** | | **Lower** | | **Upper** | |
| Age |  | S3 |  | 94 |  | 44.266 |  | 12.573 |  | 1.297 |  | 41.691 |  | 46.841 |  |
|  |  | SC |  | 70 |  | 44.614 |  | 13.392 |  | 1.601 |  | 41.421 |  | 47.807 |  |
| Anxiety |  | S3 |  | 94 |  | 5.309 |  | 3.433 |  | 0.354 |  | 4.605 |  | 6.012 |  |
|  |  | SC |  | 72 |  | 5.236 |  | 3.973 |  | 0.468 |  | 4.302 |  | 6.170 |  |
| Depression |  | S3 |  | 94 |  | 7.872 |  | 3.811 |  | 0.393 |  | 7.092 |  | 8.653 |  |
|  |  | SC |  | 72 |  | 7.361 |  | 3.186 |  | 0.375 |  | 6.612 |  | 8.110 |  |
| Loneliness |  | S3 |  | 94 |  | 21.085 |  | 13.255 |  | 1.367 |  | 18.370 |  | 23.800 |  |
|  |  | SC |  | 72 |  | 20.014 |  | 11.354 |  | 1.338 |  | 17.346 |  | 22.682 |  |
| Cognitive |  | S3 |  | 92 |  | 81.024 |  | 8.000 |  | 0.834 |  | 79.367 |  | 82.681 |  |
|  |  | SC |  | 73 |  | 79.828 |  | 8.145 |  | 0.953 |  | 77.928 |  | 81.729 |  |
| Confinement |  | S3 |  | 94 |  | -27.733 |  | 2.815 |  | 0.290 |  | -28.309 |  | -27.156 |  |
|  |  | SC |  | 73 |  | -28.397 |  | 0.602 |  | 0.070 |  | -28.538 |  | -28.257 |  |
| Spontaneous_ITI |  | S3 |  | 93 |  | 770.066 |  | 274.870 |  | 28.503 |  | 713.457 |  | 826.675 |  |
|  |  | SC |  | 73 |  | 722.990 |  | 343.650 |  | 40.221 |  | 642.810 |  | 803.169 |  |
| Paced_Asyn |  | S3 |  | 61 |  | 26.448 |  | 59.479 |  | 7.615 |  | 11.215 |  | 41.682 |  |
|  |  | SC |  | 47 |  | 3.340 |  | 119.247 |  | 17.394 |  | -31.672 |  | 38.352 |  |
| Paced_ITI |  | S3 |  | 83 |  | 971.729 |  | 67.827 |  | 7.445 |  | 956.918 |  | 986.539 |  |
|  |  | SC |  | 59 |  | 994.176 |  | 65.793 |  | 8.566 |  | 977.030 |  | 1011.322 |  |
|  | | | | | | | | | | | | | | | |
